# Implication of the intestinal microbiome as a potential surrogate marker of immune responsiveness to experimental therapies in autoimmune diabetes

**DOI:** 10.1371/journal.pone.0173968

**Published:** 2017-03-16

**Authors:** James C. Needell, Charles A. Dinarello, Diana Ir, Charles E. Robertson, Sarah M. Ryan, Miranda E. Kroehl, Daniel N. Frank, Danny Zipris

**Affiliations:** 1 Barbara Davis Center for Childhood Diabetes, University of Colorado Denver, Aurora, Colorado, United States of America; 2 Division of Infectious Diseases, University of Colorado School of Medicine, Aurora, Colorado, United States of America; 3 University of Colorado Microbiome Research Consortium (MiRC), Aurora, Colorado, United States of America; 4 Department of Biostatistics and Informatics, Colorado School of Public Health and University of Colorado Denver, Aurora, Colorado, United States of America; "INSERM", FRANCE

## Abstract

Type 1 diabetes (T1D) is an autoimmune proinflammatory disease with no effective intervention. A major obstacle in developing new immunotherapies for T1D is the lack of means for monitoring immune responsiveness to experimental therapies. The LEW1.WR1 rat develops autoimmunity following infection with the parvovirus Kilham rat virus (KRV) via mechanisms linked with activation of proinflammatory pathways and alterations in the gut bacterial composition. We used this animal to test the hypothesis that intervention with agents that block innate immunity and diabetes is associated with a shift in the gut microbiota. We observed that infection with KRV results in the induction of proinflammatory gene activation in both the spleen and pancreatic lymph nodes. Furthermore, administering animals the histone deacetylase inhibitor ITF-2357 and IL-1 receptor antagonist (Anakinra) induced differential STAT-1 and the p40 unit of IL-12/IL-23 gene expression. Sequencing of bacterial 16S rRNA genes demonstrated that both ITF-2357 and Anakinra alter microbial diversity. ITF-2357 and Anakinra modulated the abundance of 23 and 8 bacterial taxa in KRV-infected animals, respectively, of which 5 overlapped between the two agents. Lastly, principal component analysis implied that ITF-2357 and Anakinra induce distinct gut microbiomes compared with those from untreated animals or rats provided KRV only. Together, the data suggest that ITF-2357 and Anakinra differentially influence the innate immune system and the intestinal microbiota and highlight the potential use of the gut microbiome as a surrogate means of assessing anti-inflammatory immune effects in type 1 diabetes.

## Introduction

Type 1 diabetes (T1D) is a proinflammatory disorder that leads to the specific destruction of insulin producing beta cells. It is thought that a combination of both genetic and environmental factors play a key role in disease mechanisms [1]. The majority of the T1D patients develop beta-cell-specific autoantibodies before disease onset [1]. Moreover, in some patients, the appearance of autoantibodies precedes hyperglycemia by many years [1]. The period of time between seroconversion and diabetes provides an opportunity for disease prevention.

The intestinal microbiota plays an essential role in gut development, metabolism, and immunity (reviewed in refs. [2]). Emerging data from humans and animals have suggested that alterations in the gut microbiota are linked with a number of metabolic and immune disorders, including T1D [3–5]. Furthermore, humans with genetic susceptibility to islet autoimmunity have altered intestinal microbiomes [3, 5, 6], but the role of these alterations in disease pathogenesis remains to be determined [7].

One of the most critical problems that clinical trials in the field of T1D currently face is the lack of non-invasive methods for monitoring effects of experimental immunomodulatory agents [8]. Clinical trials have used a number of metabolic outcomes such as the C peptide response following a mixed-meal tolerance test, HbA1c levels, and insulin usage, to assess effects induced by experimental drugs on the disease status. However, in a significant portion of previous trials 1) clinical effects in the treatment versus the placebo groups were not detected [9, 10], 2) an effect was observed only in a subset of treated individuals [11], or 3) the effect was not durable despite continuous drug administration [12]. Therefore, there is a pressing need for new strategies, to better monitor potential responses to immunotherapies.

The LEW1.WR1 rat develops islet autoimmunity following infection with the parvovirus Kilham Rat Virus (KRV) (reviewed in ref. [13]). The diseases closely resembles the human disorder in terms of histopathology, pathogenesis, lack of sex bias, and MHC class II association [14]. Diabetes can be detected in the LEW1.WR1 rat beginning at 14 days following virus infection [15]. We recently demonstrated that the innate immune system plays a key role in the mechanism triggering beta cell autoimmunity in the BioBreeding Diabetes Resistant and LEW1.WR1 rats [4, 15–17]. Indeed, activation of the innate immune system with TLR agonists, such as the viral mimic polyinosinic: polycytidylic acid (poly I:C) or CpG DNA, followed by infection with KRV substantially exacerbates diabetes [18, 19]. Moreover, infection with KRV leads to a robust proinflammatory response linked with the up-regulation of a vast array of proinflammatory cytokines and chemokines in the spleen, pancreatic lymph nodes and Peyer’s patches via mechanisms that involve TLR9 pathways [4, 17, 18]. Lastly, modulation of virus-induced innate immunity with steroids [18], IL-1 receptor antagonist [16], histone deacetylase inhibitors [15], or antibiotics [4] ameliorates disease development.

Herein, we used the LEW1.WR1 rodent model to test the possibility that the intestinal microbiome may be used as a tool of monitoring effects induced by experimental immunotherapies. For this purpose, we analyzed the effect of histone deacetylase inhibitor ITF-2357 (reviewed in ref. [20]) and IL-1 receptor antagonist (Anakinra, reviewed in ref. [21]) on the innate immune system and intestinal microbiome. We have previously shown that treatment with Anakinra and ITF-2357 interferes with islet autoimmunity and prevents beta cell destruction [15, 16]. Our data indicate that ITF-2357 and Anakinra differentially modulate virus-induced innate immunity and the gut bacterial composition early in the disease course. The data underscore novel opportunities for using the gut microbiome as a potential tool to detect drug-induced immune effects in individuals undergoing experimental immunotherapies.

## Materials and methods

### Animals, viruses, and innate immunity blockade

**Fig 1:**
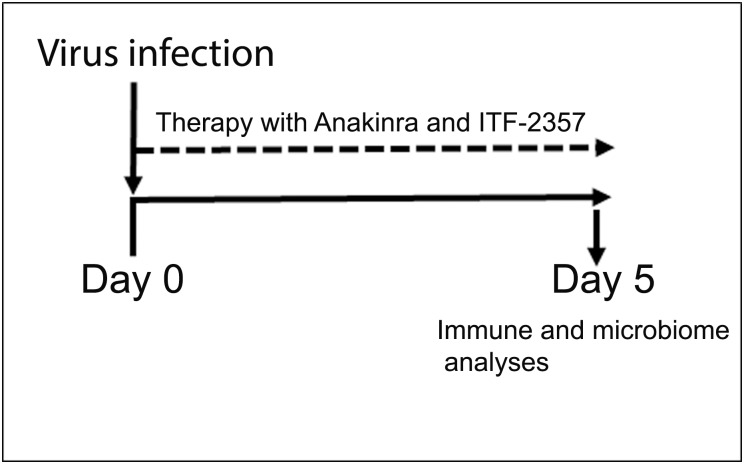
Treatment of LEW1.WR1 rats with Anakinra and ITF-2357. LEW1.WR1 rats at the age of 21–25 days were left untreated or infected with KRV. They were left with no further treatment of administered Anakinra or ITF-2357 on 5 consecutive days as described in Materials and Methods. The gut microbiome and KRV-induced innate immunity were analyzed on day 5 post-infection.

LEW1.WR1 rats were bred and housed in our specific pathogen-free facility. This study was carried out in strict accordance with the recommendations in the Guide for Care and Use of Laboratory Animals of the National Institutes of Health. The protocol was approved by the Committee on the Ethics of Animal Experiments of the University of Colorado Denver (permit number: B-79715(10)1E). To test the hypothesis that therapy with Anakinra and ITF-2357 modulate the gut microbiota, rats at the age of 21–25 days were divided into 6 control and experimental groups. Group 1 was left untreated (control uninfected). Group 2 was injected i.p. with 1 x 10^7^ PFU of KRV only, whereas Group 3 and Group 4 were i.p. injected with Anakinra on 5 consecutive days (50 μg/g body weight, obtained from Amgen, Thousand Oaks, CA) and were uninfected or infected with KRV, respectively (Fig 1). Group 5 and Group 6 were gavaged with ITF-2357 on 5 consecutive days (30 μg/g body weight, obtained from Italfarmaco, Milano, Italy) and were uninfected or KRV infected, respectively. Fecal samples were collected on day 5 post-infection and frozen immediately at -80 degrees until use.

### High-throughput DNA sequencing for microbiome analysis

Bacterial profiles were determined by broad-range analysis of 16S rRNA genes following our previously described methods [4]. In brief, DNA was extracted from fecal specimens using the QIAmp DNA stool mini kit (Qiagen, Valencia, CA). Broad-range PCR amplicons were generated using barcoded primers that target the V4 variable region of the 16S rRNA gene: primers 534F (5’ GTGCCAGCMGCCGCGGTAA) and 806R (5’ GGACTACHVGGGTWTCTAAT). PCR products were normalized using a SequalPrep^™^ kit (Invitrogen, Carlsbad, CA), pooled, and quantified by Qubit Fluorometer 2.0 (Invitrogen, Carlsbad, CA). The pool was diluted to 4nM and denatured with 0.2 N NaOH at room temperature. The denatured DNA was diluted to 15pM and spiked with 25% of the Illumina PhiX control DNA prior to loading the sequencer. Illumina paired-end sequencing was performed on the Ilumina MiSeq platform with version v2.3.0.8 of the MiSeq Control Software and version v2.3.32 of MiSeq Reporter, using a 600 cycle version 3 reagent kit. Illumina Miseq paired-end reads were aligned to Norwegian rat reference genome rn4 with bowtie2 and matching sequences discarded (ref. [22], and Homo Sapiens UCSC Hg19 Human Genome Sequence from iGenome [http://support.illumina.com/sequencing/sequencing_software/igenome.ilmn]). As previously described, the paired-end sequences were sorted by sample via barcodes in the paired reads with a python script. The sorted paired reads were assembled using phrap and pairs that did not assemble were discarded. Assembled sequence ends were trimmed over a moving window of 5 nucleotides until average quality met or exceeded 20. Trimmed sequences with more than 1 ambiguity or shorter than 150 nt were discarded. Potential chimeras identified with Uchime (usearch6.0.203_i86linux32) [23] using the Schloss [24] Silva reference sequences were removed from subsequent analyses. Assembled sequences were aligned and classified with SINA (1.3.0-r23838) [25] using the bacterial sequences in Silva 115NR99 [26] as reference configured to yield the Silva taxonomy. Operational taxonomic units (OTUs) were produced by clustering sequences with identical taxonomic assignments. Demultiplexed paired end sequence data were deposited in the NCBI Sequence Read Archive under project accession number SRP078351.

### Quantitative RT-PCR

RNA extraction, cDNA synthesis, and quantitative RT-PCR were performed as previously described [17]. The standards used for the gene amplification were TOPO plasmid vectors (Invitrogen) expressing a ∼500-bp DNA fragment derived from the mRNA sequence of the gene of interest that includes the ∼100-bp sequence used for the PCR amplification. The primers were synthesized by Integrated DNA Technologies (Coralville, IA). Their sequences have been previously published [17]. Data for gene expression in the spleen and pancreatic lymph nodes were calculated as the ratio of gene expression to β-actin expression in the same sample of cDNA.

### Statistical analysis

The Explicet software package was used for display and diversity analysis [27]. All other data analysis was performed using SAS (SAS Institute Inc., Cary, NC, USA) or R (www.cran.org) software. The relative abundance (RA) of each taxon was calculated as the number of high-quality sequences of a given taxon divided by the total number of high-quality 16S rRNA sequences in a sample. For principal component analysis (PCA) a small constant (1/total number of sequences) was added to the sequence counts and the centered log ratio transformation (CLR) was calculated. Shannon diversity, Shannon evenness, and richness (Sobs) were calculated using rarefaction and compared across groups using a Wilcoxon rank based test. To compare RA of taxa across groups, a two-stage testing process was implemented. In the first stage, an overall Wilcoxon rank based test was used to determine whether one or more groups differed significantly from the others, and therefore select taxa of interest for further comparisons. If this overall p-value was significant (p<0.05), the taxa under evaluation was included in the second stage of testing. In this stage, pairwise differences between all groups were calculated. A false discovery rate (FDR) adjustment for multiple comparisons was applied to this second stage of testing; comparisons with p<0.10 after FDR were considered significant. Differences in microbiome composition (i.e., beta-diversity) between subsets were quantified by the Bray-Curtis index using the *vegan* R package, which performs a non-parametric multivariate analysis of variance (PERMANOVA with 10,000 replicate resamplings). Statistical comparisons for the qPCR data were made using ANOVA with a Bonferroni multiple-comparison test.

## Results

### Anakinra and ITF-2357 modulate KRV-induced innate immunity in the spleen and pancreatic lymph nodes

**Fig 2:**
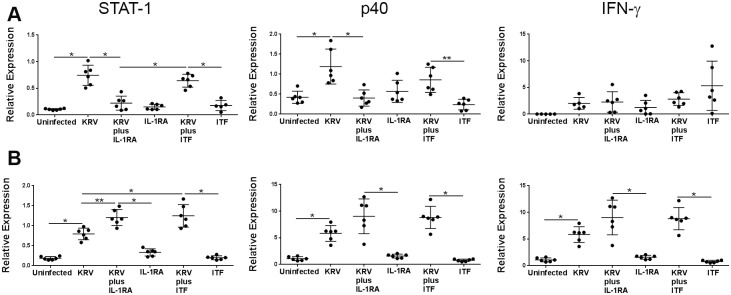
The effect of Anakinra and ITF-2357 on KRV-induced innate immunity in the spleen and pancreatic lymph nodes. LEW1.WR1 rats were left untreated, were injected with KRV, were treated with KRV plus Anakinra or ITF-2357 (ITF) beginning on the day of infection, or treated with Anakinra or ITF-2357 only as indicated in the figure. RNA was extracted from the spleen (A) and pancreatic lymph nodes (B) 5 days post-infection, and the relative expression level of the indicated genes was evaluated using quantitative RT-PCR. The results are presented as the mRNA expression of the gene of interest relative to the expression of β-actin in tissue from individual animals. Statistical analyses were performed using an ANOVA with Bonferroni's correction for multiple comparisons. *p < 0.001; **p < 0.01

We sought to investigate whether the gut microbiome could be used as a sensor of drug-induced immune modulation. To this end, we first analyzed the effect of Anakinra and ITF-2357 on virus-induced innate immunity. Animals were infected with KRV and administered Anakinra and ITF-2357 as shown in Materials and Methods and in Fig 1 (n = 6 per group). Spleens sand pancreatic lymph nodes were removed on day 5 post-infection at the time when inflammation is detectable in the spleen and pancreatic lymph nodes [18], and the expression level of transcripts for STAT-1, the p40 subunit of IL-12 and IL-23 and IFN-γ was assessed by quantitative RT-PCR. The data presented in Fig 2 indicate that infection with KRV leads to an increase in the expression level of STAT-1 and the p40 subunit of IL-12 and IL-23 but not IFN-γ in the spleen (*p* < 0.001 for both). In contrast, KRV upregulated the expression of all of these genes in pancreatic lymph nodes (*p* < 0.001 for all vs. uninfected). We further found that therapy with Anakinra but not ITF-2357 reduced STAT-1 and the p40 subunit transcript levels in the spleen (p<0.001 for both compared with KRV only). However, both Anakinra and ITF-2357 induced the expression of STAT-1 transcript levels in pancreatic lymph nodes (p<0.01 and p<0.001, respectively, versus KRV only) and moderately increased the level of the p40 subunit and IFN-γ transcripts in pancreatic lymph nodes (p>0.05). These findings suggest that Anakinra and ITF-2357 differentially modulate proinflammatory gene expression in the spleen.

### ITF-2357- and Anakinra treatment modulates the fecal microbiome

**Fig 3:**
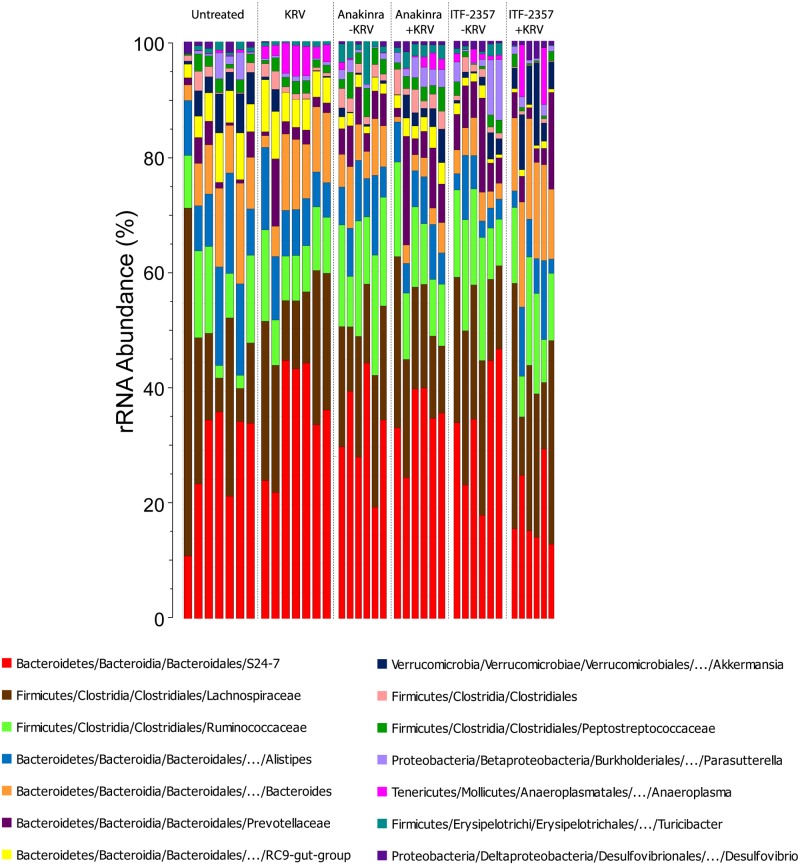
The gut microbiome in rats treated with Anakinra and ITF-2357. Stacked bar chart of median percent counts of Operational Taxonomic Units (OTU) representing bacterial genera with a frequency of ≥1% of total counts in the stool from subjects with and without islet autoimmunity as indicated in the figure. The relative abundances are inferred from 16S rRNA sequence counts in datasets. The X and Y axes represent the sample name and percentages of bacterial taxa, respectively.

We tested the hypothesis that treating LEW1.WR1 rats with the anti-inflammatory agents ITF-2357 and Anakinra would alter the composition of the intestinal microbiome. Intestinal microbiota were analyzed by bacterial 16S rRNA gene sequencing of fecal DNA on day 5 post-infection (Fig 1). A total of 130 genus-level taxa were identified within the six control and experimental rat groups (*n* = 6–7 rats per group). The highthroughput sequencing generated 1,805,321 high-quality 16S rRNA sequences (mean sequence length: 297 nt; median sequences/sample: 48,804 [range: 23,360 to 67,825]; median Goods coverage: ≥ 99.9% at the rarefaction point of 15,841). Fig 3 indicates that the intestinal microbiota of LEW1.WR1 rats were dominated by the Bacteroidetes genera *S24-7*, *Bacteroides*, and *Alistipes*, along with the Firmicutes bacterial families *Lachnospiraceae* and *Ruminococcaceae*. Furthermore, the abundance of a number of bacterial genera such as *Bacteroides*, *RC9-gut group*, and *Anaeroplasma* appeared to be different among experimental groups. These data imply that administering LEW1.WR1 rats agents that block innate immune pathways can lead to alterations in the intestinal microbiome.

### ITF-2357- and Anakinra alter bacterial biodiversity

**Fig 4:**
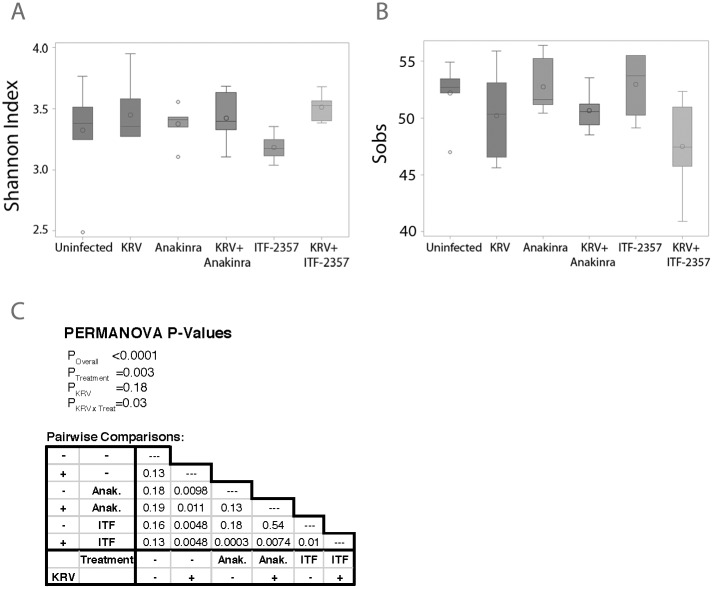
Bacterial biodiversity in animals treated with anti-inflammatory agents. Shannon alpha diversity (A) and Shannon evenness biodiversity indices (B), respectively, across the experimental groups. The area inside the box represents the interquartile range (IQR: 25th to 75th percentiles), the median and mean are denoted by a line and a circle, respectively. The whiskers extend 1.5 IQR from the box, the observations outside of this range are displayed as points. Panel C: Results of permutation-based analysis of variance (PERMANOVA) tests across all treatment groups (under the “PERMANOVA P-Values” heading) and pairs of treatment groups (table).

We next sought to assess the effect of the anti-inflammatory therapy on the gut bacterial diversity (n = 6–7 per group). Administering ITF-2357 or Anakinra to LEW1.WR1 rats significantly altered the fecal microbiota of both KRV-infected and uninfected animals. Differences in both Shannon alpha diversity (p = 0.0569; Fig 4A) and Shannon evenness (p = 0.0097; Fig 4B) were observed across the six treatment groups; in contrast, richness (Sobs) was not affected by KRV or drug treatments. Furthermore, PERMANOVA tests indicated an overall significant differences (p<0.0001) in microbiota across the treatment groups. In this analysis, infection with KRV was not significantly associated with alterations in the fecal microbiome composition, (*p* = 0.18), whereas drug treatment (*p* = 0.003) and the KRV*drug interaction (p = 0.03) were significantly linked with the gut bacterial composition (Fig 4C). These data imply that treatments with HDACi and Anakinra substantially influence taxa number and abundance as well as species evenness.

### Principal component analysis reveals three distinct clusters of animals

**Fig 5:**
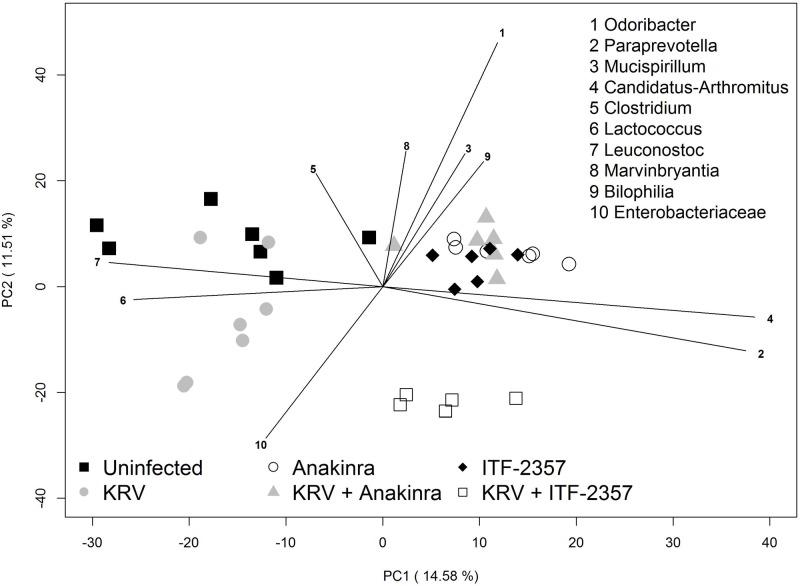
Principal component analysis of the intestinal microbiota from rats treated with innate immune blockers. The PCA scores are displayed for each sample. The different colors and shapes correspond to the various treatment groups as indicated in the figure and described in Materials and Methods. Vectors corresponding to the indicated top 10 genera with the highest loadings are displayed. The magnitude and direction correspond to the weights.

We used principal component analysis (PCA) to further corroborate and identify differences in the distributions of bacterial genera from treated and untreated animals (n = 6–7 per group). Using this approach, we detected clear separation of treatment groups along principal component axes PC1 and PC2 (Fig 5 and S1 Table). Animals that were not treated with ITF-2357 or Anakinra clustered away from treated animals, regardless of KRV infection. The animal group administered with KRV plus ITF-2357 clustered at a distinct location, whereas the remainder of the ITF-2357- or Anakinra-treated animals clustered together. Thus, similar to the PERMANOVA test, KRV infection was not associated with dramatic changes in the fecal microbiota compared with treatment with ITF-2357 or Anakinra. Biplot analysis indicated that separation along PC1 was driven by differences in the abundances of *Lactococcus* and *Leuconostoc* (enriched in animals administered or ITF-2357 or Anakinra) along with *Paraprevotella* and *Arthromitus* (enriched in animals administered ITF-2357 or Anakinra). Other organisms listed in Fig 5 were more influential in determining separation along PC2. Together, the data provide further evidence that Anakinra and ITF-2357 induce substantial shifts in the intestinal microbiome.

### Specific intestinal bacterial taxa are affected by therapy with Anakinra and ITF-2357

To identify individual taxa affected by either Anakinra or ITF-2357 therapy, relative abundances were compared across groups (n = 6–7 per group) using a Wilcoxon rank based test. A subsequent pairwise testing was conducted if a significant difference was found (p<0.05). In the overall tests, significant differences were observed in the relative abundances of 44 of the 130 total taxa (Table 1, S2 Table). Infection with KRV resulted in alterations in the abundances of 4 Firmicutes and 3 Proteobacteria taxa compared with naïve rats. ITF-2357 and Anakinra also modulated the abundances of 23 and 8 intestinal taxa in infected rats, respectively, while 5 of these taxa were altered by both drugs. Among the most notable alterations induced by Anakinra and ITF-2357 were 27-fold and 52-fold increases in the median abundance of the bacterial genus *Akkermansia*, respectively (adjusted *p* = 0.07 and *p*<0.005 vs. KRV only, respectively). ITF-2357 modulated the abundances of bacterial groups from the phyla Bacteroidetes, Firmicutes, Proteobacteria, and Actinobacteria, with the most substantial alterations observed in the levels of *Turicibacter* and *Candidatus-Arthromitus* (i.e., segmented filamentous bacteria). Similar to our previously published PCR findings [26, 27], the highthroughput data from the current study indicate that KRV increases the abundances of *Bifidobacterium* and *Clostridium* compared with the uninfected control animals, and therapies with ITF-2357 and Anakinra reduce the level of these genera compared with rats administered KRV only (data not shown); however the differences seen using the present highthroughput approach were not statistically significant probably due to the considerable differences in the size of the data sets associated with these surveys as well as the different statistical approaches used for data analysis.

**Table 1:** Median percent abundances of bacterial taxa in the gut from Anakinra- and ITF-2357-treated animals.

Taxa	Treatment					
	Uninfected^≠^	KRV	Anakinra	KRV plus Anakinra	ITF	KRV plus ITF
Bacteroidetes/S24-7*	31.06	33.13	29.30^¶^	33.28	32.25	13.94
Bacteroidetes/Butyricimonas	0.21	0.97	0.24	0.65	0.29	1.20
Bacteroidetes/Parabacteroides	1.15	1.47	0.17	0.26	0.26	2.48
Bacteroidetes/Paraprevotella	0.00	0.00	0.06	0.02	0.04	0.05
Bacteroidetes/Prevotellaceae*	1.14	1.86	4.58	5.14	5.61	2.96
Bacteroidetes/Rikenella	0.20	0.63	0.13	0.20	0.03	0.00
Bacteroidetes/Odoribacter	0.37	0.00	0.40	0.57	0.17	0.00
Bacteroidetes/Bacteroides	7.61	10.1	6.0	2.7	4.0	14.71
Verrucomicrobia/Akkermansia	3.70	0.09	0.30	2.52	1.12	4.82
Firmicutes/Marvinbryantia	0.01	0.05	0.03	0.00	0.04	0.00
Firmicutes/Clostridiales*	0.89	0.86	1.66	2.56	1.02	0.12
Firmicutes/Clostridium	0.29	0.45	0.23	0.26	0.23	0.00
Firmicutes/Erysipelotrichaceae*	0.06	0.07	0.03	0.02	0.04	0.01
Firmicutes/Candidatus-Arthromitus	0.00	0.00	0.13	0.06	0.14	1.04
Firmicutes/Leuconostoc	0.01	0.00	0.00	0.00	0.00	0.00
Firmicutes/Turicibacter	0.70	1.12	1.92	1.89	0.63	0.02
Firmicutes/Firmicutes	0.00	0.00	0.02	0.01	0.01	0.01
Firmicutes/Weissella	0.00	0.00	0.00	0.00	0.00	0.00
Firmicutes/Roseburia	0.06	0.31	0.16	0.12	0.19	0.15
Firmicutes/Lactococcus	0.01	0.01	0.00	0.00	0.00	0.00
Firmicutes/Streptococcus	0.06	0.02	0.01	0.00	0.02	0.01
Firmicutes/Blautia	0.00	0.03	0.02	0.00	0.02	0.01
Firmicutes/Pseudobutyrivibrio	0.01	0.00	0.00	0.00	0.00	0.00
Firmicutes/Ruminococcaceae*	8.39	7.11	16.23	9.91	14.38	9.76
Firmicutes/Ruminococcus	0.68	1.05	0.96	0.89	0.19	0.05
Firmicutes/RC9-gut-group	4.34	4.42	1.90	2.53	1.30	0.49
Firmicutes/Haemophilus	0.02	0.01	0.01	0.00	0.01	0.04
Firmicutes/Peptococcaceae*	0.21	0.23	0.53	0.24	0.40	1.05
Firmicutes/RF9	0.02	0.62	0.31	0.25	0.08	0.01
Firmicutes/Coprococcus	0.03	0.11	0.06	0.08	0.05	0.72
Firmicutes/Carnobacteriaceae*	0.00	0.00	0.00	0.00	0.00	0.00
Firmicutes/Anaerovorax	0.00	0.01	0.02	0.03	0.01	0.01
Firmicutes/Peptostreptococcaceae*	1.63	1.48	2.43	1.87	1.43	0.52
Proteobacteria/γ-proteobacteria*	0.00	0.00	0.00	0.00	0.00	0.00
Proteobacteria/Escherichia-Shigella	0.01	0.03	0.00	0.01	0.04	0.19
Proteobacteria/Thalassospira	0.01	0.39	0.21	0.43	0.12	0.46
Proteobacteria/Acinetobacter	0.00	0.00	0.00	0.00	0.00	0.00
Proteobacteria/Enterobacteriaceae*	0.00	0.00	0.00	0.00	0.00	0.00
Proteobacteria/Helicobacter	0.03	0.00	0.00	0.06	0.00	0.00
Proteobacteria/Bilophila	0.00	0.00	0.00	0.00	0.02	0.00
Actinobacteria/Collinsella	0.00	0.00	0.00	0.00	0.00	0.00
Actinobacteria/Bifidobacterium	0.15	0.51	0.16	0.14	0.12	0.02
Candidate-division-TM7*	0.01	0.00	0.02	0.02	0.03	0.00

^¶^The highlights in the KRV column represent significant differences compared with uninfected rats; the highlights in the Anakinra and ITF-2357 columns represent significant differences versus the uninfected control; the highlights in the KRV plus Anakinra or KRV plus ITF-2357 represent significant differences compared with KRV.

^**≠**^The levels of significance for these comparisons are indicated in S2 Table.

*Entry represents a higher-order group of taxa that could not be assigned to finer levels of taxonomic resolution. All other taxa represent genus-level classifications.

Finally, ITF-2357 and Anakinra modulated the abundances of more than 20 taxa in the intestine of uninfected animals, of which 11 overlapped between the two agents. Collectively, the data suggest that therapies with either Anakinra or ITF-2357 differentially modulate the abundances of individual bacterial taxa.

## Discussion

Numerous clinical trials have been conducted in the last twenty years to treat T1D, however the disease remains without known etiology or effective immunotherapeutic interventions [28]. One of the major reasons for this failure is the lack of suitable means to monitor effects of experimental immunotherapies on the immune system. Indeed, there is a consensus that new non-invasive clinical means are urgently needed to promote the discovery of new interventions for T1D [28]. Importantly, data indicating whether a potential immunotherapy can modulate immunity are crucial even if the trial itself fails to show clinical efficacy [28]. Herein, we used the LEW1.WR1 rat model of virus-induced inflammation and diabetes to test the effect of anti-inflammatory therapies with Anakinra and ITF-2357 on innate immunity and the intestinal microbiome shortly after commencing the therapy. We present data indicating that in addition to altering the innate immune system, intervention with Anakinra and ITF-2357 differentially modulates the gut bacterial structure as reflected by shifts in the overall gut bacterial microbiomes and bacterial diversity and alterations in the abundance of individual bacterial taxa, most notably those of the Bacteroidetes phylum. Based on these findings, we propose that alterations in the intestinal microbiota could potentially be used as a surrogate tool to detect immune responsiveness to experimental immunotherapies.

How therapy with Anakinra or ITF-2357 modulates the level of commensal bacteria is unclear. In addition, it remains to be identified whether the affected gut microbes are directly linked with mechanisms of autoimmunity. Owing to the interdependence between the immune system and the gut microbiota [29, 30], we postulate that the differential effect exerted by Anakinra and ITF-2357 on the gut microbiome might be associated at least in part with different effects they have on the immune system. Indeed, data from the present and our earlier studies [15, 16] are in accordance with the possibility that Anakinra and ITF-2357 induce a differential effect on the KRV-induced innate immunity. This difference could be linked with the fact that Anakinra inhibits the IL-1 signaling pathway by binding to the IL-1R1 [31], whereas ITF-2357 suppresses class I and class II histone deacetylases and may therefore induce broader effects on immunity [32]. In any case, both Anakinra and ITF-2357 were observed to alter cytokine expression in various experimental systems [33, 34]. Additional studies are necessary to establish whether a cause-and-effect relationship exists between ITF-2357- and Anakinra-induced alterations in innate immunity and the gut bacterial composition.

One of the most dramatic alterations induced by therapies with ITF-2357 and Anakinra was a 30-50-fold increase in the median abundance of the Verrucomicrobia genus *Akkermansia* that was modulated by both drugs. It may be hypothesized that this increase is associated with potential anti-inflammatory effects induced by ITF-2357 and Anakinra. Indeed, the level of the *Akkermansia* species *A*. *muciniphila*, a mucin-degrading bacteria residing in the mucus layer was reported to induce a beneficial effect on diabetes in NOD mice [35]. Another interesting result was the ITF-2357-induced increase in the abundance of the Firmicutes taxon *Candidatus-Arthromitus*. *Candidatus-Arthromitus* is the segmented filamentous bacteria (SFB) that has been associated with both diabetes induction and prevention (reviewed in ref. [36]). Finally, we observed that therapy with ITF-2357 led to an increase in the abundance of a number of Bacteroidetes taxa including the *Bacteroides* genus. This shift could promote an anti-inflammatory environment, since *Bacteroides* can induce Treg cells and anti-inflammatory cytokine expression and inhibit NF-κB signaling pathways [37] although some members of the *Bacteroides* genus were also shown to promote pro-inflammatory pathways in humans and mice [38]. Whether alterations in the abundances of the above taxa could be useful in assessing drug-induced immune modulation in subjects undergoing anti-inflammatory therapies remains to be determined in future clinical trials. In any case, this possibility is consistent with data from other disorders. For example, a previous report demonstrated a correlation between a shift in the gut microbiota and the response to steroid treatments in individuals with ulcerative colitis [39]. Another study provided evidence for alterations in the gut microbiome from subjects using metformin and statins [40]. Indeed, subjects who used metformin had an increased abundance of *Escherichia coli* and a positively correlated with pathways associated with the degradation and utilization of D-glucarate and D-galactarate and pyruvate fermentation [40].

Why Anakinra and ITF-2357 induce a differential effect on the spleen versus pancreatic lymph nodes is unclear but could be linked with differences in the composition and function that exist between these lymphoid organs [41]. It remains to be determined whether and how innate pathways affected by Anakinra- and ITF-2357 are directly linked to mechanisms of islet autoimmunity. Likewise, whether Anakinra- and ITF-2357-induced alterations in the abundance of gut bacteria can be used as a biomarker of therapy outcome awaits further investigation.

In conclusion, we show that two different anti-inflammatory drugs that modulate innate immunity and the gut microbiome and innate immunity in diabetes-susceptible animals. These observations have important clinical implication as they point to the possibility that the gut microbiome could potentially be harnessed as a novel means to facilitate drug discovery in T1D and potentially other autoimmune disorders.

## Supporting information

S1 Table The contribution of taxa to the components of the PCA. (DOCX)

S2 Table Results from Wilcoxon tests indicating the pairwise comparisons for taxa with a statistically significant difference across groups (overall p-value). (DOCX)

## References

[1] Gianani R, Eisenbarth GS. The stages of type 1A diabetes: 2005. Immunol Rev. 2005;204:232–49. 10.1111/j.0105-2896.2005.00248.x 15790362

[2] Caballero S, Pamer EG. Microbiota-Mediated Inflammation and Antimicrobial Defense in the Intestine. Annu Rev Immunol. 2015;33(1):227–56. 25581310 10.1146/annurev-immunol-032713-120238 PMC4540477

[3] Alkanani AK, Hara N, Gottlieb PA, Ir D, Robertson CE, Wagner BD, et al Alterations in Intestinal Microbiota Correlate with Susceptibility to Type 1 Diabetes. Diabetes. 2015. 10.2337/db14-1847 PMC4587635 26068542

[4] Hara N, Alkanani AK, Ir D, Robertson CE, Wagner BD, Frank DN, et al Prevention of Virus-Induced Type 1 Diabetes with Antibiotic Therapy. J Immunol. 2012;189(8):3805–14. 10.4049/jimmunol.1201257 22988033

[5] Wen L, Ley RE, Volchkov PY, Stranges PB, Avanesyan L, Stonebraker AC, et al Innate immunity and intestinal microbiota in the development of Type 1 diabetes. Nature. 2008;455(7216):1109–13. 10.1038/nature07336 18806780 PMC2574766

[6] Vaarala O, Atkinson MA, Neu J. The "perfect storm" for type 1 diabetes: the complex interplay between intestinal microbiota, gut permeability, and mucosal immunity. Diabetes. 2008;57(10):2555–62. 10.2337/db08-0331 18820210 PMC2551660

[7] Frank DN, Zhu W, Sartor RB, Li E. Investigating the biological and clinical significance of human dysbioses. Trends Microbiol. 2011;19(9):427–34. 10.1016/j.tim.2011.06.005 21775143 PMC3164499

[8] Tooley JE, Herold KC. Biomarkers in type 1 diabetes: application to the clinical trial setting. Current Opinion in Endocrinology, Diabetes and Obesity. 2014;21(4):287–92. 10.1097/MED.0000000000000076 PMC4163003 24937037

[9] Mandrup-Poulsen T, Pickersgill L, Donath MY. Blockade of interleukin 1 in type 1 diabetes mellitus. Nat Rev Endocrinol. 2010;6(3):158–66. 10.1038/nrendo.2009.271 20173777

[10] Moran A, Bundy B, Becker DJ, DiMeglio LA, Gitelman SE, Goland R, et al Interleukin-1 antagonism in type 1 diabetes of recent onset: two multicentre, randomised, double-blind, placebo-controlled trials. The Lancet. 2013;38(9881):1905–15. 10.1016/S0140-6736(13)60023-9 PMC3827771 23562090

[11] Wherrett DK, Bundy B, Becker DJ, DiMeglio LA, Gitelman SE, Goland R, et al Antigen-based therapy with glutamic acid decarboxylase (GAD) vaccine in patients with recent-onset type 1 diabetes: a randomised double-blind trial. The Lancet. 2011;378(9788):319–27. 10.1016/S0140-6736(11)60895-7 PMC3580128 21714999

[12] Atkinson MA, Eisenbarth GS, Michels AW. Type 1 diabetes. The Lancet. 383(9911):69–82. 10.1016/S0140-6736(13)60591-7. PMC4380133 23890997

[13] Mordes JP, Bortell R, Blankenhorn EP, Rossini AA, Greiner DL. Rat models of type 1 diabetes: genetics, environment, and autoimmunity. ILAR J. 2004;45(3):278–91. 15229375 10.1093/ilar.45.3.278

[14] Qaisar N, Lin S, Ryan G, Yang C, Oikemus SR, Brodsky MH, et al A Critical Role for the Type I Interferon Receptor in Virus-Induced Autoimmune Diabetes in Rats. Diabetes. 2017;66(1):145–57. 10.2337/db16-0462 27999109 PMC5204313

[15] Hara N, Alkanani A, Dinarello C, Zipris D. Histone deacetylase inhibitor suppresses virus-induced proinflammatory responses and type 1 diabetes. J Mol Med. 2013:1–10. 23982318 10.1007/s00109-013-1078-1

[16] Hara N, Alkanani AK, Dinarello CA, Zipris D. Modulation of virus-induced innate immunity and type 1 diabetes by IL-1 blockade. Innate Immun. 2014;20(6):574–84. 10.1177/1753425913502242 24062197

[17] Wolter TR, Wong R, Sarkar SA, Zipris D. DNA microarray analysis for the identification of innate immune pathways implicated in virus-induced autoimmune diabetes. Clin Immunol. 2009;132(1):103–15. 10.1016/j.clim.2009.02.007 19328037

[18] Londono P, Komura A, Hara N, Zipris D. Brief dexamethasone treatment during acute infection prevents virus-induced autoimmune diabetes. Clin Immunol. 2010;135(3):401–11. 10.1016/j.clim.2010.01.007 20167539

[19] Zipris D, Lien E, Xie JX, Greiner DL, Mordes JP, Rossini AA. TLR Activation Synergizes with Kilham Rat Virus Infection to Induce Diabetes in BBDR Rats. J Immunol. 2005;174(1):131–42. 15611235 10.4049/jimmunol.174.1.131

[20] Furlan A, Monzani V, Reznikov LL, Leoni F, Fossati G, Modena D, et al Pharmacokinetics, safety and inducible cytokine responses during a phase 1 trial of the oral histone deacetylase inhibitor ITF2357 (givinostat). Mol Med. 2011;17(5–6):353–62. 10.2119/molmed.2011.00020 21365126 PMC3105139

[21] Dinarello CA, Simon A, Van der Meer JWM. Treating inflammation by blocking interleukin-1 in a broad spectrum of diseases. Nat Rev Drug Discov. 2012;11(8):633–52. 10.1038/nrd3800 22850787 PMC3644509

[22] Langmead B, Salzberg SL. Fast gapped-read alignment with Bowtie 2. Nat Meth. 2012;9(4):357–9. http://www.nature.com/nmeth/journal/v9/n4/abs/nmeth.1923.html#supplementary-information. 10.1038/nmeth.1923 PMC3322381 22388286

[23] Edgar RC, Haas BJ, Clemente JC, Quince C, Knight R. UCHIME improves sensitivity and speed of chimera detection. Bioinformatics. 2011;27(16):2194–200. 10.1093/bioinformatics/btr381 21700674 PMC3150044

[24] Schloss PD, Westcott SL. Assessing and improving methods used in operational taxonomic unit-based approaches for 16S rRNA gene sequence analysis. Appl Environ Microbiol. 2011;77(10):3219–26. 10.1128/AEM.02810-10 21421784 PMC3126452

[25] Pruesse E, Peplies J, Glockner FO. SINA: accurate high-throughput multiple sequence alignment of ribosomal RNA genes. Bioinformatics. 2012;28(14):1823–9. 10.1093/bioinformatics/bts252 22556368 PMC3389763

[26] Quast C, Pruesse E, Yilmaz P, Gerken J, Schweer T, Yarza P, et al The SILVA ribosomal RNA gene database project: improved data processing and web-based tools. Nucleic Acids Res. 2013;41(Database issue):D590–D6. 10.1093/nar/gks1219 23193283 PMC3531112

[27] Robertson CE, Harris JK, Wagner BD, Granger D, Browne K, Tatem B, et al Explicet: graphical user interface software for metadata-driven management, analysis and visualization of microbiome data. Bioinformatics. 2013;29(23):3100–1. 10.1093/bioinformatics/btt526 24021386 PMC3834795

[28] Roep BO, Peakman M. Surrogate end points in the design of immunotherapy trials: emerging lessons from type 1 diabetes. Nat Rev Immunol. 2010;10(2):145–52. 10.1038/nri2705 20098462

[29] Hooper LV, Macpherson AJ. Immune adaptations that maintain homeostasis with the intestinal microbiota. Nat Rev Immunol. 2010;10(3):159–69. 10.1038/nri2710 20182457

[30] Thaiss CA, Levy M, Suez J, Elinav E. The interplay between the innate immune system and the microbiota. Curr Opin Immunol. 2014;26:41–8. 10.1016/j.coi.2013.10.016. 24556399

[31] Gabay C, Lamacchia C, Palmer G. IL-1 pathways in inflammation and human diseases. Nat Rev Rheumatol. 2010;6(4):232–41. 10.1038/nrrheum.2010.4 20177398

[32] Dinarello CA. Inhibitors of histone deacetylases as anti-inflammatory drugs. Ernst Schering Res Found Workshop. 2006;(56):45–60. 16331856 10.1007/3-540-37673-9_3

[33] Dinarello CA. Interleukin-1 in the pathogenesis and treatment of inflammatory diseases. Blood. 2011;117(14):3720–32. 10.1182/blood-2010-07-273417 21304099 PMC3083294

[34] Dinarello CA, Fossati G, Mascagni P. Histone deacetylase inhibitors for treating a spectrum of diseases not related to cancer. Mol Med. 2011;17(5–6):333–52. 10.2119/molmed.2011.00116 21556484 PMC3105126

[35] Everard A, Belzer C, Geurts L, Ouwerkerk JP, Druart C, Bindels LB, et al Cross-talk between Akkermansia muciniphila and intestinal epithelium controls diet-induced obesity. Proc Natl Acad Sci. 2013;110:9066–71. 10.1073/pnas.1219451110 23671105 PMC3670398

[36] David LA, Maurice CF, Carmody RN, Gootenberg DB, Button JE, Wolfe BE, et al Diet rapidly and reproducibly alters the human gut microbiome. Nature. 2014;505(7484):559–63. 10.1038/nature12820 24336217 PMC3957428

[37] Maynard CL, Elson CO, Hatton RD, Weaver CT. Reciprocal interactions of the intestinal microbiota and immune system. Nature. 2012;489(7415):231–41. 10.1038/nature11551 22972296 PMC4492337

[38] Chervonsky AV. Intestinal commensals: influence on immune system and tolerance to pathogens. Curr Opin Immunol. 2012;24(3):255–60. 10.1016/j.coi.2012.03.002 22445718

[39] Michail S, Durbin M, Turner D, Griffiths AM, Mack DR, Hyams J, et al Alterations in the gut microbiome of children with severe ulcerative colitis. Inflamm Bowel Dis. 2012;18(10):1799–808. 10.1002/ibd.22860 22170749 PMC3319508

[40] Zhernakova A, Kurilshikov A, Bonder MJ, Tigchelaar EF, Schirmer M, Vatanen T, et al Population-based metagenomics analysis reveals markers for gut microbiome composition and diversity. Science. 2016;352(6285):565–9. 10.1126/science.aad3369 27126040 PMC5240844

[41] Mebius RE, Kraal G. Structure and function of the spleen. Nat Rev Immunol. 2005;5(8):606–16. 10.1038/nri1669 16056254

