# Supplementary material for: Implication of the intestinal microbiome as a potential surrogate marker of immune responsiveness to experimental therapies in autoimmune diabetes
Source: PLoS One. 2017 Mar 16;12(3):e0173968. doi: 10.1371/journal.pone.0173968 (PMC5354421; doi:10.1371/journal.pone.0173968)
Supplement: S1 Table — (DOCX) [file pone.0173968.s001.docx]

**S1 Table.** The contribution of taxa

to the components of the PCA

|  | **PC1** | **PC2** |
| --- | --- | --- |
| Atopobium | 0.008931 | -0.07354 |
| Bifidobacteriaceae | -0.00107 | -0.02758 |
| Bifidobacterium | -0.05973 | 0.070375 |
| Collinsella | 0.093557 | 0.067471 |
| Coriobacteriaceae | -0.0853 | 0.127973 |
| Corynebacterium | 0.020738 | -0.07506 |
| Curtobacterium | -0.00107 | -0.02758 |
| Enterorhabdus | -0.064 | 0.105571 |
| Gaiella | -0.00107 | -0.02758 |
| Rothia | -0.1484 | 0.059097 |
| Alistipes | -0.00588 | 0.001759 |
| Bacteroidales | 0.016481 | 0.009901 |
| Bacteroides | -0.00606 | -0.04369 |
| Bacteroidetes | 0.01778 | 0.025948 |
| Barnesiella | 0.089301 | -0.02309 |
| Butyricimonas | -0.01274 | -0.03979 |
| Chitinophagaceae | 0.022954 | -0.01542 |
| Chryseobacterium | -0.029 | 0.005342 |
| Flavobacterium | -0.00107 | -0.02758 |
| Odoribacter | 0.124766 | 0.485252 |
| Parabacteroides | -0.03169 | -0.06435 |
| Paraprevotella | 0.395094 | -0.12768 |
| Porphyromonadaceae | -0.00307 | 0.022584 |
| Prevotella | 0.027304 | 0.00223 |
| Prevotellaceae | 0.047308 | -0.00999 |
| RC9-gut-group | -0.03251 | 0.021405 |
| Rikenella | -0.08258 | 0.138846 |
| Rikenellaceae | -0.00107 | -0.02758 |
| S24-7 | 0.00648 | -0.00053 |
| VC2.1-Bac22 | 0.029807 | 0.048671 |
| Candidate-division-OD1 | 0.001614 | -0.07013 |
| Candidate-division-TM7 | 0.149779 | 0.138903 |
| OPB56 | 0.016664 | -0.01573 |
| 4C0d-2 | 0.111186 | -0.05184 |
| Chloroplast | 0.148124 | 0.056644 |
| Mucispirillum | 0.089455 | 0.264149 |
| Aerococcaceae | 0.004667 | 0.00375 |
| Allobaculum | -0.04136 | 0.039909 |
| Anaerofilum | 0.016138 | -0.10232 |
| Anaerofustis | 0.028774 | -0.02873 |
| Anaerostipes | -0.03084 | -0.06139 |
| Anaerotruncus | 0.027637 | -0.02401 |
| Anaerovorax- | 0.111207 | -0.05886 |
| Bacilli | -0.07869 | 0.021609 |
| Blautia | 0.009509 | -0.04594 |
| Butyrivibrio | -0.02012 | -0.01501 |
| Candidatus-Arthromitus | 0.404579 | -0.06065 |
| Carnobacteriaceae | 0.162792 | 0.053167 |
| Catenibacterium | -0.00107 | -0.02758 |
| Christensenella | -0.00566 | -0.03283 |
| Christensenellaceae | -0.00563 | 0.019092 |
| Clostridiales | 0.024552 | 0.042975 |
| Clostridium | -0.07268 | 0.224978 |
| Coprococcus | 0.022667 | -0.05594 |
| Dialister | -0.00107 | -0.02758 |
| Dorea | -0.14595 | 0.016692 |
| Enterococcaceae | -0.00107 | -0.02758 |
| Enterococcus | -0.00286 | -0.00695 |
| Erysipelotrichaceae | -0.03473 | 0.1015 |
| Faecalibacterium | 0.059271 | -0.05581 |
| Family-XIII-Incertae-Sedis | -0.0027 | 0.008504 |
| Firmicutes | 0.177799 | 0.060851 |
| Flavonifractor | 0.006853 | 0.030683 |
| Gemella | -0.00107 | -0.02758 |
| Globicatella | -0.01816 | -0.02432 |
| Gracilibacteraceae | -0.00107 | -0.02758 |
| Granulicatella | 0.04339 | 0.020419 |
| Hydrogenoanaerobacterium | -6.17E-02 | -0.10243 |
| Lachnospira | 0.072397 | 0.0676 |
| Lachnospiraceae | 0.016005 | -0.0082 |
| Lactobacillaceae | 0.001614 | -0.07013 |
| Lactobacillales | -0.04627 | -0.01297 |
| Lactobacillus | -0.01716 | -0.01365 |
| Lactococcus | -0.2709 | -0.02595 |
| Leuconostoc | -0.29786 | 0.047846 |
| Marvinbryantia | 0.025217 | 0.269149 |
| Oscillibacter | 0.028855 | -0.01131 |
| Oscillospira | 0.091175 | -0.07781 |
| Oxobacter | -0.00107 | -0.02758 |
| Peptococcaceae | 0.026934 | -0.02956 |
| Peptococcus | 0.033034 | -0.00014 |
| Peptostreptococcaceae | 0.006845 | 0.012233 |
| Phascolarctobacterium | -0.00107 | -0.02758 |
| Pseudobutyrivibrio | -0.17557 | 0.112943 |
| Roseburia | 0.014913 | -0.02697 |
| Ruminococcaceae | 0.032143 | -0.0079 |
| Ruminococcus | -0.01873 | 0.054221 |
| Staphylococcus | -0.06644 | -0.02525 |
| Streptococcaceae | -0.04795 | -0.00436 |
| Streptococcus | -0.08015 | -0.0176 |
| Subdoligranulum | 0.01064 | -0.02861 |
| Turicibacter | -0.01163 | 0.088465 |
| Veillonella | -0.05263 | -0.00204 |
| Veillonellaceae | -0.00107 | -0.02758 |
| Weissella | -0.1415 | 0.042598 |
| Fusobacterium | 0.044151 | 0.015352 |
| Acetobacter | -0.02262 | -0.04691 |
| Acinetobacter | -0.19034 | 0.04189 |
| Actinobacillus | -0.02145 | -0.00866 |
| Aeromonas | -0.04795 | -0.00436 |
| Arcobacter | -0.00107 | -0.02758 |
| Bilophila | 0.109734 | 0.249028 |
| Comamonadaceae | -0.06667 | 0.012428 |
| Comamonas | -0.0683 | -0.02664 |
| Delftia | -0.00107 | -0.02758 |
| Desulfovibrio | 0.031462 | -0.02731 |
| Enhydrobacter | -0.02992 | -0.00968 |
| Enterobacter | -0.12457 | 0.055563 |
| Enterobacteriaceae | -0.12706 | -0.30167 |
| Escherichia-Shi | 0.044223 | -0.18319 |
| Gammaproteobacteria | 0.01306 | -0.1171 |
| Haemophilus | -0.08606 | -0.11575 |
| Helicobacter | -0.06949 | 0.120411 |
| Hydrogenophaga | -0.00107 | -0.02758 |
| Massilia | -0.00107 | -0.02758 |
| Neisseriaceae | -0.029 | 0.005342 |
| Parasutterella | 0.017821 | 0.002111 |
| Pasteurella | -0.04671 | -0.04414 |
| Pasteurellaceae | -0.00721 | -0.0836 |
| Pectobacterium | -0.04795 | -0.00436 |
| Proteobacteria | 0.002456 | -0.06503 |
| Proteus | 0.045792 | -0.19491 |
| Ralstonia | 0.010089 | -0.07008 |
| Roseomonas | -0.00327 | -0.00917 |
| Sutterella | -0.00107 | -0.02758 |
| Thalassospira | 0.048195 | -0.06091 |
| mitochondria | 0.099566 | -0.07199 |
| Anaeroplasma | -0.13892 | -0.03874 |
| RF9 | 0.013607 | 0.054088 |
| Akkermansia | 0.098582 | 0.081698 |
