# Supplementary material for: Implication of the intestinal microbiome as a potential surrogate marker of immune responsiveness to experimental therapies in autoimmune diabetes
Source: PLoS One. 2017 Mar 16;12(3):e0173968. doi: 10.1371/journal.pone.0173968 (PMC5354421; doi:10.1371/journal.pone.0173968)
Supplement: S2 Table — (DOCX) [file pone.0173968.s002.docx]

|  |  |  |  |  |
| --- | --- | --- | --- | --- |
| **S2 Table.** Results from Wilcoxon tests indicating the pairwise comparisons for the genera with a statistically significant difference across groups (overall p-value). | | | | |
| **Taxa** | **Overall p-value** | **Comparison** | **p-value** | **FDR p-value** |
| RC9-gut-group | <.001 | ITF vs KRV + ITF | 0.592 | 0.725 |
|  |  | Anakinra vs ITF | <.001 | <.001 |
|  |  | Anakinra vs KRV + Anakinra | <.001 | <.001 |
|  |  | KRV vs ITF | <.001 | <.001 |
|  |  | KRV vs Anakinra | <.001 | 0.003 |
|  |  | KRV vs KRV + ITF | <.001 | <.001 |
|  |  | KRV vs KRV + Anakinra | 0.009 | 0.034 |
|  |  | Uninfected vs ITF | <.001 | <.001 |
|  |  | Uninfected vs Anakinra | 0.016 | 0.052 |
|  |  | Uninfected vs KRV + ITF | <.001 | <.001 |
|  |  | Uninfected vs KRV + Anakinra | <.001 | 0.002 |
|  |  | Uninfected vs KRV | 0.145 | 0.267 |
|  |  | KRV + Anakinra vs KRV + ITF | 0.001 | 0.009 |
| Candidatus-Arthromitus | <.001 | ITF vs KRV + ITF | 0.024 | 0.068 |
|  |  | Anakinra vs ITF | <.001 | <.001 |
|  |  | Anakinra vs KRV + Anakinra | <.001 | <.001 |
|  |  | KRV vs ITF | <.001 | <.001 |
|  |  | KRV vs Anakinra | <.001 | 0.006 |
|  |  | KRV vs KRV + ITF | 0.002 | 0.011 |
|  |  | KRV vs KRV + Anakinra | 0.074 | 0.155 |
|  |  | Uninfected vs ITF | <.001 | <.001 |
|  |  | Uninfected vs Anakinra | 0.004 | 0.018 |
|  |  | Uninfected vs KRV + ITF | <.001 | 0.004 |
|  |  | Uninfected vs KRV + Anakinra | 0.022 | 0.065 |
|  |  | Uninfected vs KRV | 0.576 | 0.711 |
|  |  | KRV + Anakinra vs KRV + ITF | 0.147 | 0.27 |
| Candidate-division-TM7 | <.001 | ITF vs KRV + ITF | 0.003 | 0.016 |
|  |  | Anakinra vs ITF | 0.166 | 0.294 |
|  |  | Anakinra vs KRV + Anakinra | <.001 | <.001 |
|  |  | KRV vs ITF | <.001 | <.001 |
|  |  | KRV vs Anakinra | 0.002 | 0.01 |
|  |  | KRV vs KRV + ITF | 0.062 | 0.136 |
|  |  | KRV vs KRV + Anakinra | 0.17 | 0.297 |
|  |  | Uninfected vs ITF | <.001 | <.001 |
|  |  | Uninfected vs Anakinra | <.001 | <.001 |
|  |  | Uninfected vs KRV + ITF | 0.001 | 0.009 |
|  |  | Uninfected vs KRV + Anakinra | 0.896 | 0.981 |
|  |  | Uninfected vs KRV | 0.135 | 0.251 |
|  |  | KRV + Anakinra vs KRV + ITF | 0.002 | 0.011 |
| Parabacteroides | <.001 | ITF vs KRV + ITF | 0.043 | 0.104 |
|  |  | Anakinra vs ITF | 0.706 | 0.832 |
|  |  | Anakinra vs KRV + Anakinra | <.001 | <.001 |
|  |  | KRV vs ITF | <.001 | <.001 |
|  |  | KRV vs Anakinra | <.001 | <.001 |
|  |  | KRV vs KRV + ITF | <.001 | 0.006 |
|  |  | KRV vs KRV + Anakinra | 0.3 | 0.449 |
|  |  | Uninfected vs ITF | <.001 | <.001 |
|  |  | Uninfected vs Anakinra | <.001 | <.001 |
|  |  | Uninfected vs KRV + ITF | 0.015 | 0.048 |
|  |  | Uninfected vs KRV + Anakinra | 0.967 | 1 |
|  |  | Uninfected vs KRV | 0.281 | 0.427 |
|  |  | KRV + Anakinra vs KRV + ITF | 0.013 | 0.045 |
| Odoribacter | <.001 | ITF vs KRV + ITF | 0.001 | 0.009 |
|  |  | Anakinra vs ITF | 0.501 | 0.644 |
|  |  | Anakinra vs KRV + Anakinra | <.001 | <.001 |
|  |  | KRV vs ITF | <.001 | <.001 |
|  |  | KRV vs Anakinra | <.001 | <.001 |
|  |  | KRV vs KRV + ITF | 0.131 | 0.246 |
|  |  | KRV vs KRV + Anakinra | 0.935 | 1 |
|  |  | Uninfected vs ITF | <.001 | 0.006 |
|  |  | Uninfected vs Anakinra | <.001 | 0.002 |
|  |  | Uninfected vs KRV + ITF | 0.758 | 0.876 |
|  |  | Uninfected vs KRV + Anakinra | 0.211 | 0.348 |
|  |  | Uninfected vs KRV | 0.242 | 0.385 |
|  |  | KRV + Anakinra vs KRV + ITF | 0.112 | 0.217 |
| Leuconostoc | <.001 | ITF vs KRV + ITF | 0.041 | 0.101 |
|  |  | Anakinra vs ITF | 0.002 | 0.011 |
|  |  | Anakinra vs KRV + Anakinra | 1 | 1 |
|  |  | KRV vs ITF | 0.002 | 0.011 |
|  |  | KRV vs Anakinra | 1 | 1 |
|  |  | KRV vs KRV + ITF | <.001 | <.001 |
|  |  | KRV vs KRV + Anakinra | 1 | 1 |
|  |  | Uninfected vs ITF | 0.002 | 0.011 |
|  |  | Uninfected vs Anakinra | 1 | 1 |
|  |  | Uninfected vs KRV + ITF | <.001 | <.001 |
|  |  | Uninfected vs KRV + Anakinra | 1 | 1 |
|  |  | Uninfected vs KRV | 1 | 1 |
|  |  | KRV + Anakinra vs KRV + ITF | <.001 | <.001 |
| Paraprevotella | <.001 | ITF vs KRV + ITF | 0.152 | 0.272 |
|  |  | Anakinra vs ITF | 0.001 | 0.009 |
|  |  | Anakinra vs KRV + Anakinra | 0.041 | 0.101 |
|  |  | KRV vs ITF | <.001 | 0.003 |
|  |  | KRV vs Anakinra | 0.566 | 0.7 |
|  |  | KRV vs KRV + ITF | <.001 | <.001 |
|  |  | KRV vs KRV + Anakinra | 0.011 | 0.039 |
|  |  | Uninfected vs ITF | 0.008 | 0.034 |
|  |  | Uninfected vs Anakinra | 0.491 | 0.641 |
|  |  | Uninfected vs KRV + ITF | <.001 | 0.002 |
|  |  | Uninfected vs KRV + Anakinra | 0.162 | 0.289 |
|  |  | Uninfected vs KRV | 0.211 | 0.348 |
|  |  | KRV + Anakinra vs KRV + ITF | 0.01 | 0.038 |
| Rikenella | <.001 | ITF vs KRV + ITF | 0.004 | 0.02 |
|  |  | Anakinra vs ITF | <.001 | <.001 |
|  |  | Anakinra vs KRV + Anakinra | 0.002 | 0.012 |
|  |  | KRV vs ITF | 0.009 | 0.035 |
|  |  | KRV vs Anakinra | <.001 | 0.007 |
|  |  | KRV vs KRV + ITF | 0.859 | 0.956 |
|  |  | KRV vs KRV + Anakinra | 0.764 | 0.88 |
|  |  | Uninfected vs ITF | <.001 | 0.001 |
|  |  | Uninfected vs Anakinra | 0.053 | 0.122 |
|  |  | Uninfected vs KRV + ITF | 0.141 | 0.261 |
|  |  | Uninfected vs KRV + Anakinra | 0.194 | 0.33 |
|  |  | Uninfected vs KRV | 0.113 | 0.217 |
|  |  | KRV + Anakinra vs KRV + ITF | 0.894 | 0.98 |
| Bifidobacterium | <.001 | ITF vs KRV + ITF | <.001 | 0.002 |
|  |  | Anakinra vs ITF | <.001 | <.001 |
|  |  | Anakinra vs KRV + Anakinra | 0.023 | 0.068 |
|  |  | KRV vs ITF | 0.007 | 0.03 |
|  |  | KRV vs Anakinra | 0.003 | 0.013 |
|  |  | KRV vs KRV + ITF | 0.216 | 0.353 |
|  |  | KRV vs KRV + Anakinra | 0.379 | 0.537 |
|  |  | Uninfected vs ITF | <.001 | 0.002 |
|  |  | Uninfected vs Anakinra | 0.072 | 0.153 |
|  |  | Uninfected vs KRV + ITF | 0.84 | 0.943 |
|  |  | Uninfected vs KRV + Anakinra | 0.606 | 0.737 |
|  |  | Uninfected vs KRV | 0.167 | 0.295 |
|  |  | KRV + Anakinra vs KRV + ITF | 0.739 | 0.86 |
| Akkermansia | <.001 | ITF vs KRV + ITF | <.001 | 0.001 |
|  |  | Anakinra vs ITF | <.001 | <.001 |
|  |  | Anakinra vs KRV + Anakinra | 0.044 | 0.106 |
|  |  | KRV vs ITF | 0.586 | 0.722 |
|  |  | KRV vs Anakinra | <.001 | 0.001 |
|  |  | KRV vs KRV + ITF | <.001 | 0.005 |
|  |  | KRV vs KRV + Anakinra | 0.025 | 0.071 |
|  |  | Uninfected vs ITF | 0.033 | 0.086 |
|  |  | Uninfected vs Anakinra | 0.008 | 0.032 |
|  |  | Uninfected vs KRV + ITF | 0.039 | 0.099 |
|  |  | Uninfected vs KRV + Anakinra | 0.468 | 0.613 |
|  |  | Uninfected vs KRV | 0.116 | 0.222 |
|  |  | KRV + Anakinra vs KRV + ITF | 0.175 | 0.302 |
| Bacteroides | <.001 | ITF vs KRV + ITF | 0.973 | 1 |
|  |  | Anakinra vs ITF | 0.026 | 0.073 |
|  |  | Anakinra vs KRV + Anakinra | <.001 | <.001 |
|  |  | KRV vs ITF | 0.449 | 0.598 |
|  |  | KRV vs Anakinra | 0.005 | 0.025 |
|  |  | KRV vs KRV + ITF | 0.468 | 0.613 |
|  |  | KRV vs KRV + Anakinra | 0.012 | 0.042 |
|  |  | Uninfected vs ITF | 0.013 | 0.045 |
|  |  | Uninfected vs Anakinra | <.001 | <.001 |
|  |  | Uninfected vs KRV + ITF | 0.014 | 0.047 |
|  |  | Uninfected vs KRV + Anakinra | 0.388 | 0.541 |
|  |  | Uninfected vs KRV | 0.084 | 0.171 |
|  |  | KRV + Anakinra vs KRV + ITF | 0.001 | 0.009 |
| Turicibacter | <.001 | ITF vs KRV + ITF | 0.221 | 0.357 |
|  |  | Anakinra vs ITF | <.001 | 0.005 |
|  |  | Anakinra vs KRV + Anakinra | <.001 | <.001 |
|  |  | KRV vs ITF | 0.152 | 0.272 |
|  |  | KRV vs Anakinra | <.001 | <.001 |
|  |  | KRV vs KRV + ITF | 0.012 | 0.041 |
|  |  | KRV vs KRV + Anakinra | 1 | 1 |
|  |  | Uninfected vs ITF | 0.325 | 0.48 |
|  |  | Uninfected vs Anakinra | 0.01 | 0.038 |
|  |  | Uninfected vs KRV + ITF | 0.843 | 0.943 |
|  |  | Uninfected vs KRV + Anakinra | 0.023 | 0.068 |
|  |  | Uninfected vs KRV | 0.023 | 0.068 |
|  |  | KRV + Anakinra vs KRV + ITF | 0.012 | 0.041 |
| Clostridiales | <.001 | ITF vs KRV + ITF | 0.873 | 0.965 |
|  |  | Anakinra vs ITF | 0.012 | 0.041 |
|  |  | Anakinra vs KRV + Anakinra | <.001 | <.001 |
|  |  | KRV vs ITF | 0.04 | 0.1 |
|  |  | KRV vs Anakinra | <.001 | <.001 |
|  |  | KRV vs KRV + ITF | 0.056 | 0.125 |
|  |  | KRV vs KRV + Anakinra | 0.512 | 0.653 |
|  |  | Uninfected vs ITF | 0.45 | 0.598 |
|  |  | Uninfected vs Anakinra | 0.002 | 0.013 |
|  |  | Uninfected vs KRV + ITF | 0.546 | 0.679 |
|  |  | Uninfected vs KRV + Anakinra | 0.055 | 0.124 |
|  |  | Uninfected vs KRV | 0.194 | 0.33 |
|  |  | KRV + Anakinra vs KRV + ITF | 0.012 | 0.041 |
| Haemophilus | <.001 | ITF vs KRV + ITF | 0.041 | 0.101 |
|  |  | Anakinra vs ITF | 0.024 | 0.07 |
|  |  | Anakinra vs KRV + Anakinra | <.001 | <.001 |
|  |  | KRV vs ITF | 0.728 | 0.854 |
|  |  | KRV vs Anakinra | 0.014 | 0.046 |
|  |  | KRV vs KRV + ITF | 0.023 | 0.067 |
|  |  | KRV vs KRV + Anakinra | 0.02 | 0.062 |
|  |  | Uninfected vs ITF | 0.864 | 0.957 |
|  |  | Uninfected vs Anakinra | 0.043 | 0.104 |
|  |  | Uninfected vs KRV + ITF | 0.07 | 0.149 |
|  |  | Uninfected vs KRV + Anakinra | 0.006 | 0.026 |
|  |  | Uninfected vs KRV | 0.617 | 0.747 |
|  |  | KRV + Anakinra vs KRV + ITF | <.001 | <.001 |
| Firmicutes | <.001 | ITF vs KRV + ITF | 0.565 | 0.7 |
|  |  | Anakinra vs ITF | 0.26 | 0.404 |
|  |  | Anakinra vs KRV + Anakinra | 0.01 | 0.039 |
|  |  | KRV vs ITF | <.001 | 0.004 |
|  |  | KRV vs Anakinra | 0.01 | 0.037 |
|  |  | KRV vs KRV + ITF | 0.002 | 0.01 |
|  |  | KRV vs KRV + Anakinra | 0.972 | 1 |
|  |  | Uninfected vs ITF | <.001 | 0.006 |
|  |  | Uninfected vs Anakinra | 0.02 | 0.062 |
|  |  | Uninfected vs KRV + ITF | 0.004 | 0.019 |
|  |  | Uninfected vs KRV + Anakinra | 0.782 | 0.896 |
|  |  | Uninfected vs KRV | 0.756 | 0.875 |
|  |  | KRV + Anakinra vs KRV + ITF | 0.002 | 0.011 |
| Escherichia-Shi | <.001 | ITF vs KRV + ITF | 0.949 | 1 |
|  |  | Anakinra vs ITF | <.001 | 0.006 |
|  |  | Anakinra vs KRV + Anakinra | <.001 | 0.001 |
|  |  | KRV vs ITF | 0.085 | 0.173 |
|  |  | KRV vs Anakinra | <.001 | <.001 |
|  |  | KRV vs KRV + ITF | 0.096 | 0.189 |
|  |  | KRV vs KRV + Anakinra | 0.374 | 0.535 |
|  |  | Uninfected vs ITF | 0.383 | 0.539 |
|  |  | Uninfected vs Anakinra | 0.009 | 0.035 |
|  |  | Uninfected vs KRV + ITF | 0.351 | 0.508 |
|  |  | Uninfected vs KRV + Anakinra | 0.106 | 0.207 |
|  |  | Uninfected vs KRV | 0.015 | 0.049 |
|  |  | KRV + Anakinra vs KRV + ITF | 0.441 | 0.593 |
| Clostridium | <.001 | ITF vs KRV + ITF | 0.054 | 0.122 |
|  |  | Anakinra vs ITF | <.001 | <.001 |
|  |  | Anakinra vs KRV + Anakinra | <.001 | 0.005 |
|  |  | KRV vs ITF | 0.028 | 0.078 |
|  |  | KRV vs Anakinra | 0.003 | 0.018 |
|  |  | KRV vs KRV + ITF | 0.713 | 0.839 |
|  |  | KRV vs KRV + Anakinra | 0.497 | 0.643 |
|  |  | Uninfected vs ITF | 0.061 | 0.134 |
|  |  | Uninfected vs Anakinra | 0.001 | 0.009 |
|  |  | Uninfected vs KRV + ITF | 0.988 | 1 |
|  |  | Uninfected vs KRV + Anakinra | 0.734 | 0.857 |
|  |  | Uninfected vs KRV | 0.734 | 0.857 |
|  |  | KRV + Anakinra vs KRV + ITF | 0.735 | 0.858 |
| Erysipelotrichaceae | <.001 | ITF vs KRV + ITF | 0.306 | 0.455 |
|  |  | Anakinra vs ITF | <.001 | <.001 |
|  |  | Anakinra vs KRV + Anakinra | 0.043 | 0.104 |
|  |  | KRV vs ITF | 0.01 | 0.037 |
|  |  | KRV vs Anakinra | 0.027 | 0.076 |
|  |  | KRV vs KRV + ITF | 0.089 | 0.179 |
|  |  | KRV vs KRV + Anakinra | 0.839 | 0.943 |
|  |  | Uninfected vs ITF | 0.049 | 0.114 |
|  |  | Uninfected vs Anakinra | 0.005 | 0.025 |
|  |  | Uninfected vs KRV + ITF | 0.302 | 0.45 |
|  |  | Uninfected vs KRV + Anakinra | 0.383 | 0.539 |
|  |  | Uninfected vs KRV | 0.501 | 0.644 |
|  |  | KRV + Anakinra vs KRV + ITF | 0.058 | 0.128 |
| Bilophila | <.001 | ITF vs KRV + ITF | 0.119 | 0.226 |
|  |  | Anakinra vs ITF | 0.541 | 0.677 |
|  |  | Anakinra vs KRV + Anakinra | 0.029 | 0.079 |
|  |  | KRV vs ITF | 0.022 | 0.065 |
|  |  | KRV vs Anakinra | 0.006 | 0.027 |
|  |  | KRV vs KRV + ITF | 0.387 | 0.541 |
|  |  | KRV vs KRV + Anakinra | 0.527 | 0.665 |
|  |  | Uninfected vs ITF | <.001 | 0.001 |
|  |  | Uninfected vs Anakinra | <.001 | <.001 |
|  |  | Uninfected vs KRV + ITF | 0.006 | 0.026 |
|  |  | Uninfected vs KRV + Anakinra | 0.013 | 0.043 |
|  |  | Uninfected vs KRV | 0.053 | 0.122 |
|  |  | KRV + Anakinra vs KRV + ITF | 0.833 | 0.941 |
| RF9 | <.001 | ITF vs KRV + ITF | 0.009 | 0.034 |
|  |  | Anakinra vs ITF | <.001 | <.001 |
|  |  | Anakinra vs KRV + Anakinra | <.001 | 0.004 |
|  |  | KRV vs ITF | 0.175 | 0.302 |
|  |  | KRV vs Anakinra | <.001 | 0.007 |
|  |  | KRV vs KRV + ITF | 0.204 | 0.339 |
|  |  | KRV vs KRV + Anakinra | 0.788 | 0.901 |
|  |  | Uninfected vs ITF | 0.011 | 0.041 |
|  |  | Uninfected vs Anakinra | 0.024 | 0.068 |
|  |  | Uninfected vs KRV + ITF | 0.996 | 1 |
|  |  | Uninfected vs KRV + Anakinra | 0.137 | 0.255 |
|  |  | Uninfected vs KRV | 0.219 | 0.357 |
|  |  | KRV + Anakinra vs KRV + ITF | 0.124 | 0.235 |
| Butyricimonas | <.001 | ITF vs KRV + ITF | <.001 | 0.007 |
|  |  | Anakinra vs ITF | 0.3 | 0.449 |
|  |  | Anakinra vs KRV + Anakinra | 0.443 | 0.595 |
|  |  | KRV vs ITF | <.001 | 0.003 |
|  |  | KRV vs Anakinra | 0.006 | 0.026 |
|  |  | KRV vs KRV + ITF | 0.542 | 0.677 |
|  |  | KRV vs KRV + Anakinra | 0.038 | 0.097 |
|  |  | Uninfected vs ITF | <.001 | 0.002 |
|  |  | Uninfected vs Anakinra | 0.004 | 0.02 |
|  |  | Uninfected vs KRV + ITF | 0.455 | 0.601 |
|  |  | Uninfected vs KRV + Anakinra | 0.028 | 0.077 |
|  |  | Uninfected vs KRV | 0.893 | 0.98 |
|  |  | KRV + Anakinra vs KRV + ITF | 0.113 | 0.217 |
| Thalassospira | <.001 | ITF vs KRV + ITF | <.001 | <.001 |
|  |  | Anakinra vs ITF | 0.591 | 0.725 |
|  |  | Anakinra vs KRV + Anakinra | 0.921 | 1 |
|  |  | KRV vs ITF | 0.083 | 0.171 |
|  |  | KRV vs Anakinra | 0.239 | 0.382 |
|  |  | KRV vs KRV + ITF | 0.011 | 0.041 |
|  |  | KRV vs KRV + Anakinra | 0.203 | 0.338 |
|  |  | Uninfected vs ITF | 0.032 | 0.085 |
|  |  | Uninfected vs Anakinra | 0.112 | 0.217 |
|  |  | Uninfected vs KRV + ITF | 0.032 | 0.085 |
|  |  | Uninfected vs KRV + Anakinra | 0.093 | 0.186 |
|  |  | Uninfected vs KRV | 0.668 | 0.795 |
|  |  | KRV + Anakinra vs KRV + ITF | <.001 | 0.003 |
| Coprococcus | <.001 | ITF vs KRV + ITF | 0.002 | 0.012 |
|  |  | Anakinra vs ITF | 0.15 | 0.272 |
|  |  | Anakinra vs KRV + Anakinra | 0.051 | 0.118 |
|  |  | KRV vs ITF | 0.145 | 0.267 |
|  |  | KRV vs Anakinra | 0.007 | 0.031 |
|  |  | KRV vs KRV + ITF | 0.093 | 0.186 |
|  |  | KRV vs KRV + Anakinra | 0.412 | 0.56 |
|  |  | Uninfected vs ITF | 0.021 | 0.064 |
|  |  | Uninfected vs Anakinra | <.001 | 0.006 |
|  |  | Uninfected vs KRV + ITF | 0.43 | 0.582 |
|  |  | Uninfected vs KRV + Anakinra | 0.093 | 0.186 |
|  |  | Uninfected vs KRV | 0.376 | 0.535 |
|  |  | KRV + Anakinra vs KRV + ITF | 0.014 | 0.047 |
| Ruminococcus | <.001 | ITF vs KRV + ITF | 0.333 | 0.488 |
|  |  | Anakinra vs ITF | <.001 | 0.001 |
|  |  | Anakinra vs KRV + Anakinra | 0.001 | 0.01 |
|  |  | KRV vs ITF | 0.633 | 0.76 |
|  |  | KRV vs Anakinra | <.001 | 0.005 |
|  |  | KRV vs KRV + ITF | 0.647 | 0.773 |
|  |  | KRV vs KRV + Anakinra | 0.742 | 0.863 |
|  |  | Uninfected vs ITF | 0.006 | 0.026 |
|  |  | Uninfected vs Anakinra | 0.164 | 0.291 |
|  |  | Uninfected vs KRV + ITF | 0.052 | 0.12 |
|  |  | Uninfected vs KRV + Anakinra | 0.048 | 0.113 |
|  |  | Uninfected vs KRV | 0.023 | 0.068 |
|  |  | KRV + Anakinra vs KRV + ITF | 0.907 | 0.989 |
| Helicobacter | 0.002 | ITF vs KRV + ITF | <.001 | 0.006 |
|  |  | Anakinra vs ITF | 0.052 | 0.12 |
|  |  | Anakinra vs KRV + Anakinra | 0.377 | 0.535 |
|  |  | KRV vs ITF | 0.761 | 0.878 |
|  |  | KRV vs Anakinra | 0.032 | 0.085 |
|  |  | KRV vs KRV + ITF | <.001 | 0.004 |
|  |  | KRV vs KRV + Anakinra | 0.004 | 0.018 |
|  |  | Uninfected vs ITF | 0.045 | 0.106 |
|  |  | Uninfected vs Anakinra | 0.945 | 1 |
|  |  | Uninfected vs KRV + ITF | 0.136 | 0.254 |
|  |  | Uninfected vs KRV + Anakinra | 0.414 | 0.562 |
|  |  | Uninfected vs KRV | 0.027 | 0.076 |
|  |  | KRV + Anakinra vs KRV + ITF | 0.508 | 0.649 |
| Prevotellaceae | 0.002 | ITF vs KRV + ITF | 0.639 | 0.765 |
|  |  | Anakinra vs ITF | 0.263 | 0.408 |
|  |  | Anakinra vs KRV + Anakinra | 0.199 | 0.333 |
|  |  | KRV vs ITF | 0.014 | 0.047 |
|  |  | KRV vs Anakinra | 0.169 | 0.297 |
|  |  | KRV vs KRV + ITF | 0.005 | 0.021 |
|  |  | KRV vs KRV + Anakinra | 0.924 | 1 |
|  |  | Uninfected vs ITF | 0.002 | 0.011 |
|  |  | Uninfected vs Anakinra | 0.037 | 0.095 |
|  |  | Uninfected vs KRV + ITF | <.001 | 0.005 |
|  |  | Uninfected vs KRV + Anakinra | 0.394 | 0.545 |
|  |  | Uninfected vs KRV | 0.448 | 0.598 |
|  |  | KRV + Anakinra vs KRV + ITF | 0.006 | 0.026 |
| Marvinbryantia | 0.002 | ITF vs KRV + ITF | 0.452 | 0.599 |
|  |  | Anakinra vs ITF | 0.002 | 0.012 |
|  |  | Anakinra vs KRV + Anakinra | 0.261 | 0.405 |
|  |  | KRV vs ITF | 0.517 | 0.656 |
|  |  | KRV vs Anakinra | <.001 | 0.005 |
|  |  | KRV vs KRV + ITF | 0.175 | 0.302 |
|  |  | KRV vs KRV + Anakinra | 0.011 | 0.039 |
|  |  | Uninfected vs ITF | 0.606 | 0.737 |
|  |  | Uninfected vs Anakinra | <.001 | 0.006 |
|  |  | Uninfected vs KRV + ITF | 0.219 | 0.357 |
|  |  | Uninfected vs KRV + Anakinra | 0.015 | 0.048 |
|  |  | Uninfected vs KRV | 0.898 | 0.981 |
|  |  | KRV + Anakinra vs KRV + ITF | 0.164 | 0.291 |
| Peptococcaceae | 0.002 | ITF vs KRV + ITF | 0.793 | 0.903 |
|  |  | Anakinra vs ITF | 0.001 | 0.009 |
|  |  | Anakinra vs KRV + Anakinra | 0.005 | 0.023 |
|  |  | KRV vs ITF | 0.011 | 0.039 |
|  |  | KRV vs Anakinra | 0.451 | 0.598 |
|  |  | KRV vs KRV + ITF | 0.006 | 0.026 |
|  |  | KRV vs KRV + Anakinra | 0.031 | 0.083 |
|  |  | Uninfected vs ITF | 0.101 | 0.199 |
|  |  | Uninfected vs Anakinra | 0.09 | 0.18 |
|  |  | Uninfected vs KRV + ITF | 0.061 | 0.134 |
|  |  | Uninfected vs KRV + Anakinra | 0.212 | 0.349 |
|  |  | Uninfected vs KRV | 0.331 | 0.487 |
|  |  | KRV + Anakinra vs KRV + ITF | 0.538 | 0.675 |
| Weissella | 0.003 | ITF vs KRV + ITF | 0.006 | 0.026 |
|  |  | Anakinra vs ITF | 0.396 | 0.545 |
|  |  | Anakinra vs KRV + Anakinra | 1 | 1 |
|  |  | KRV vs ITF | 0.396 | 0.545 |
|  |  | KRV vs Anakinra | 1 | 1 |
|  |  | KRV vs KRV + ITF | <.001 | 0.006 |
|  |  | KRV vs KRV + Anakinra | 1 | 1 |
|  |  | Uninfected vs ITF | 0.396 | 0.545 |
|  |  | Uninfected vs Anakinra | 1 | 1 |
|  |  | Uninfected vs KRV + ITF | <.001 | 0.006 |
|  |  | Uninfected vs KRV + Anakinra | 1 | 1 |
|  |  | Uninfected vs KRV | 1 | 1 |
|  |  | KRV + Anakinra vs KRV + ITF | <.001 | 0.006 |
| Roseburia | 0.003 | ITF vs KRV + ITF | <.001 | 0.003 |
|  |  | Anakinra vs ITF | 0.112 | 0.217 |
|  |  | Anakinra vs KRV + Anakinra | 0.196 | 0.331 |
|  |  | KRV vs ITF | 0.077 | 0.161 |
|  |  | KRV vs Anakinra | 0.851 | 0.952 |
|  |  | KRV vs KRV + ITF | 0.04 | 0.099 |
|  |  | KRV vs KRV + Anakinra | 0.266 | 0.411 |
|  |  | Uninfected vs ITF | 0.498 | 0.643 |
|  |  | Uninfected vs Anakinra | 0.368 | 0.529 |
|  |  | Uninfected vs KRV + ITF | 0.002 | 0.013 |
|  |  | Uninfected vs KRV + Anakinra | 0.033 | 0.085 |
|  |  | Uninfected vs KRV | 0.279 | 0.426 |
|  |  | KRV + Anakinra vs KRV + ITF | 0.339 | 0.494 |
| Anaerovorax- | 0.004 | ITF vs KRV + ITF | 0.931 | 1 |
|  |  | Anakinra vs ITF | 0.604 | 0.737 |
|  |  | Anakinra vs KRV + Anakinra | 0.008 | 0.033 |
|  |  | KRV vs ITF | 0.064 | 0.139 |
|  |  | KRV vs Anakinra | 0.188 | 0.32 |
|  |  | KRV vs KRV + ITF | 0.076 | 0.159 |
|  |  | KRV vs KRV + Anakinra | 0.151 | 0.272 |
|  |  | Uninfected vs ITF | 0.545 | 0.679 |
|  |  | Uninfected vs Anakinra | 0.282 | 0.427 |
|  |  | Uninfected vs KRV + ITF | 0.492 | 0.641 |
|  |  | Uninfected vs KRV + Anakinra | <.001 | 0.004 |
|  |  | Uninfected vs KRV | 0.02 | 0.062 |
|  |  | KRV + Anakinra vs KRV + ITF | 0.002 | 0.012 |
| Anaeroplasma | 0.005 | ITF vs KRV + ITF | 0.002 | 0.01 |
|  |  | Anakinra vs ITF | 0.007 | 0.028 |
|  |  | Anakinra vs KRV + Anakinra | 0.52 | 0.658 |
|  |  | KRV vs ITF | <.001 | 0.003 |
|  |  | KRV vs Anakinra | 0.247 | 0.392 |
|  |  | KRV vs KRV + ITF | 0.422 | 0.572 |
|  |  | KRV vs KRV + Anakinra | 0.077 | 0.161 |
|  |  | Uninfected vs ITF | 0.054 | 0.122 |
|  |  | Uninfected vs Anakinra | 0.392 | 0.545 |
|  |  | Uninfected vs KRV + ITF | 0.199 | 0.333 |
|  |  | Uninfected vs KRV + Anakinra | 0.83 | 0.941 |
|  |  | Uninfected vs KRV | 0.049 | 0.114 |
|  |  | KRV + Anakinra vs KRV + ITF | 0.286 | 0.432 |
| Lactococcus | 0.006 | ITF vs KRV + ITF | 0.881 | 0.971 |
|  |  | Anakinra vs ITF | 0.026 | 0.074 |
|  |  | Anakinra vs KRV + Anakinra | 0.498 | 0.643 |
|  |  | KRV vs ITF | 0.003 | 0.013 |
|  |  | KRV vs Anakinra | 0.367 | 0.528 |
|  |  | KRV vs KRV + ITF | 0.004 | 0.018 |
|  |  | KRV vs KRV + Anakinra | 0.821 | 0.933 |
|  |  | Uninfected vs ITF | 0.199 | 0.333 |
|  |  | Uninfected vs Anakinra | 0.334 | 0.488 |
|  |  | Uninfected vs KRV + ITF | 0.253 | 0.399 |
|  |  | Uninfected vs KRV + Anakinra | 0.105 | 0.207 |
|  |  | Uninfected vs KRV | 0.067 | 0.145 |
|  |  | KRV + Anakinra vs KRV + ITF | 0.007 | 0.029 |
| Streptococcus | 0.009 | ITF vs KRV + ITF | 0.258 | 0.403 |
|  |  | Anakinra vs ITF | 0.502 | 0.644 |
|  |  | Anakinra vs KRV + Anakinra | 0.041 | 0.102 |
|  |  | KRV vs ITF | 0.121 | 0.229 |
|  |  | KRV vs Anakinra | 0.385 | 0.541 |
|  |  | KRV vs KRV + ITF | 0.011 | 0.04 |
|  |  | KRV vs KRV + Anakinra | 0.222 | 0.358 |
|  |  | Uninfected vs ITF | 0.409 | 0.558 |
|  |  | Uninfected vs Anakinra | 0.88 | 0.971 |
|  |  | Uninfected vs KRV + ITF | 0.061 | 0.134 |
|  |  | Uninfected vs KRV + Anakinra | 0.057 | 0.127 |
|  |  | Uninfected vs KRV | 0.472 | 0.617 |
|  |  | KRV + Anakinra vs KRV + ITF | <.001 | 0.004 |
| Family-XIII-Incertae-Sedis | 0.01 | ITF vs KRV + ITF | 0.046 | 0.11 |
|  |  | Anakinra vs ITF | 0.177 | 0.303 |
|  |  | Anakinra vs KRV + Anakinra | 0.002 | 0.013 |
|  |  | KRV vs ITF | 0.619 | 0.747 |
|  |  | KRV vs Anakinra | 0.079 | 0.163 |
|  |  | KRV vs KRV + ITF | 0.146 | 0.268 |
|  |  | KRV vs KRV + Anakinra | 0.148 | 0.27 |
|  |  | Uninfected vs ITF | 0.619 | 0.747 |
|  |  | Uninfected vs Anakinra | 0.403 | 0.553 |
|  |  | Uninfected vs KRV + ITF | 0.018 | 0.057 |
|  |  | Uninfected vs KRV + Anakinra | 0.02 | 0.062 |
|  |  | Uninfected vs KRV | 0.34 | 0.494 |
|  |  | KRV + Anakinra vs KRV + ITF | 0.961 | 1 |
| Enterobacteriaceae | 0.011 | ITF vs KRV + ITF | 0.381 | 0.539 |
|  |  | Anakinra vs ITF | 0.404 | 0.553 |
|  |  | Anakinra vs KRV + Anakinra | 0.002 | 0.013 |
|  |  | KRV vs ITF | 0.015 | 0.048 |
|  |  | KRV vs Anakinra | 0.002 | 0.013 |
|  |  | KRV vs KRV + ITF | 0.094 | 0.187 |
|  |  | KRV vs KRV + Anakinra | 1 | 1 |
|  |  | Uninfected vs ITF | 0.177 | 0.303 |
|  |  | Uninfected vs Anakinra | 0.039 | 0.099 |
|  |  | Uninfected vs KRV + ITF | 0.601 | 0.735 |
|  |  | Uninfected vs KRV + Anakinra | 0.258 | 0.403 |
|  |  | Uninfected vs KRV | 0.258 | 0.403 |
|  |  | KRV + Anakinra vs KRV + ITF | 0.094 | 0.187 |
| Pseudobutyrivibrio | 0.013 | ITF vs KRV + ITF | 0.043 | 0.104 |
|  |  | Anakinra vs ITF | 0.235 | 0.376 |
|  |  | Anakinra vs KRV + Anakinra | 0.888 | 0.977 |
|  |  | KRV vs ITF | 0.28 | 0.426 |
|  |  | KRV vs Anakinra | 0.916 | 0.997 |
|  |  | KRV vs KRV + ITF | 0.004 | 0.019 |
|  |  | KRV vs KRV + Anakinra | 0.972 | 1 |
|  |  | Uninfected vs ITF | 0.208 | 0.345 |
|  |  | Uninfected vs Anakinra | 0.944 | 1 |
|  |  | Uninfected vs KRV + ITF | 0.002 | 0.013 |
|  |  | Uninfected vs KRV + Anakinra | 0.833 | 0.941 |
|  |  | Uninfected vs KRV | 0.86 | 0.956 |
|  |  | KRV + Anakinra vs KRV + ITF | 0.004 | 0.02 |
| Oscillibacter | 0.013 | ITF vs KRV + ITF | 0.069 | 0.147 |
|  |  | Anakinra vs ITF | 0.002 | 0.012 |
|  |  | Anakinra vs KRV + Anakinra | 0.087 | 0.177 |
|  |  | KRV vs ITF | 0.001 | 0.008 |
|  |  | KRV vs Anakinra | 0.835 | 0.942 |
|  |  | KRV vs KRV + ITF | 0.089 | 0.18 |
|  |  | KRV vs KRV + Anakinra | 0.057 | 0.126 |
|  |  | Uninfected vs ITF | 0.035 | 0.091 |
|  |  | Uninfected vs Anakinra | 0.276 | 0.424 |
|  |  | Uninfected vs KRV + ITF | 0.702 | 0.829 |
|  |  | Uninfected vs KRV + Anakinra | 0.515 | 0.654 |
|  |  | Uninfected vs KRV | 0.197 | 0.331 |
|  |  | KRV + Anakinra vs KRV + ITF | 0.768 | 0.883 |
| Desulfovibrio | 0.021 | ITF vs KRV + ITF | 0.039 | 0.098 |
|  |  | Anakinra vs ITF | <.001 | 0.006 |
|  |  | Anakinra vs KRV + Anakinra | 0.028 | 0.077 |
|  |  | KRV vs ITF | 0.288 | 0.434 |
|  |  | KRV vs Anakinra | 0.014 | 0.047 |
|  |  | KRV vs KRV + ITF | 0.329 | 0.484 |
|  |  | KRV vs KRV + Anakinra | 0.77 | 0.884 |
|  |  | Uninfected vs ITF | 0.048 | 0.113 |
|  |  | Uninfected vs Anakinra | 0.108 | 0.211 |
|  |  | Uninfected vs KRV + ITF | 0.99 | 1 |
|  |  | Uninfected vs KRV + Anakinra | 0.521 | 0.659 |
|  |  | Uninfected vs KRV | 0.352 | 0.508 |
|  |  | KRV + Anakinra vs KRV + ITF | 0.497 | 0.643 |
| Blautia | 0.022 | ITF vs KRV + ITF | 0.004 | 0.018 |
|  |  | Anakinra vs ITF | 0.155 | 0.277 |
|  |  | Anakinra vs KRV + Anakinra | 0.22 | 0.357 |
|  |  | KRV vs ITF | 0.532 | 0.669 |
|  |  | KRV vs Anakinra | 0.433 | 0.584 |
|  |  | KRV vs KRV + ITF | 0.023 | 0.068 |
|  |  | KRV vs KRV + Anakinra | 0.049 | 0.114 |
|  |  | Uninfected vs ITF | 0.512 | 0.653 |
|  |  | Uninfected vs Anakinra | 0.45 | 0.598 |
|  |  | Uninfected vs KRV + ITF | 0.025 | 0.07 |
|  |  | Uninfected vs KRV + Anakinra | 0.052 | 0.12 |
|  |  | Uninfected vs KRV | 0.977 | 1 |
|  |  | KRV + Anakinra vs KRV + ITF | 0.79 | 0.902 |
| Acinetobacter | 0.025 | ITF vs KRV + ITF | 0.832 | 0.941 |
|  |  | Anakinra vs ITF | 0.032 | 0.085 |
|  |  | Anakinra vs KRV + Anakinra | 1 | 1 |
|  |  | KRV vs ITF | 0.032 | 0.085 |
|  |  | KRV vs Anakinra | 1 | 1 |
|  |  | KRV vs KRV + ITF | 0.02 | 0.062 |
|  |  | KRV vs KRV + Anakinra | 1 | 1 |
|  |  | Uninfected vs ITF | 0.032 | 0.085 |
|  |  | Uninfected vs Anakinra | 1 | 1 |
|  |  | Uninfected vs KRV + ITF | 0.02 | 0.062 |
|  |  | Uninfected vs KRV + Anakinra | 1 | 1 |
|  |  | Uninfected vs KRV | 1 | 1 |
|  |  | KRV + Anakinra vs KRV + ITF | 0.02 | 0.062 |
| Carnobacteriaceae | 0.029 | ITF vs KRV + ITF | 1 | 1 |
|  |  | Anakinra vs ITF | 1 | 1 |
|  |  | Anakinra vs KRV + Anakinra | 0.04 | 0.099 |
|  |  | KRV vs ITF | 0.117 | 0.223 |
|  |  | KRV vs Anakinra | 0.131 | 0.245 |
|  |  | KRV vs KRV + ITF | 0.117 | 0.223 |
|  |  | KRV vs KRV + Anakinra | 0.558 | 0.693 |
|  |  | Uninfected vs ITF | 0.014 | 0.046 |
|  |  | Uninfected vs Anakinra | 0.017 | 0.055 |
|  |  | Uninfected vs KRV + ITF | 0.014 | 0.046 |
|  |  | Uninfected vs KRV + Anakinra | 0.716 | 0.841 |
|  |  | Uninfected vs KRV | 0.344 | 0.499 |
|  |  | KRV + Anakinra vs KRV + ITF | 0.033 | 0.086 |
| Alistipes | 0.032 | ITF vs KRV + ITF | 0.172 | 0.301 |
|  |  | Anakinra vs ITF | 0.283 | 0.429 |
|  |  | Anakinra vs KRV + Anakinra | 0.863 | 0.957 |
|  |  | KRV vs ITF | 0.983 | 1 |
|  |  | KRV vs Anakinra | 0.292 | 0.438 |
|  |  | KRV vs KRV + ITF | 0.196 | 0.331 |
|  |  | KRV vs KRV + Anakinra | 0.376 | 0.535 |
|  |  | Uninfected vs ITF | 0.042 | 0.103 |
|  |  | Uninfected vs Anakinra | 0.332 | 0.487 |
|  |  | Uninfected vs KRV + ITF | 0.002 | 0.01 |
|  |  | Uninfected vs KRV + Anakinra | 0.255 | 0.401 |
|  |  | Uninfected vs KRV | 0.048 | 0.113 |
|  |  | KRV + Anakinra vs KRV + ITF | 0.031 | 0.084 |
| Peptostreptococcaceae | 0.033 | ITF vs KRV + ITF | 0.75 | 0.87 |
|  |  | Anakinra vs ITF | 0.149 | 0.272 |
|  |  | Anakinra vs KRV + Anakinra | 0.024 | 0.068 |
|  |  | KRV vs ITF | 0.036 | 0.092 |
|  |  | KRV vs Anakinra | 0.001 | 0.009 |
|  |  | KRV vs KRV + ITF | 0.069 | 0.147 |
|  |  | KRV vs KRV + Anakinra | 0.255 | 0.401 |
|  |  | Uninfected vs ITF | 0.855 | 0.953 |
|  |  | Uninfected vs Anakinra | 0.222 | 0.358 |
|  |  | Uninfected vs KRV + ITF | 0.625 | 0.752 |
|  |  | Uninfected vs KRV + Anakinra | 0.267 | 0.412 |
|  |  | Uninfected vs KRV | 0.029 | 0.079 |
|  |  | KRV + Anakinra vs KRV + ITF | 0.502 | 0.644 |
| Gammaproteobacteria | 0.039 | ITF vs KRV + ITF | 1 | 1 |
|  |  | Anakinra vs ITF | 0.006 | 0.026 |
|  |  | Anakinra vs KRV + Anakinra | 0.008 | 0.032 |
|  |  | KRV vs ITF | 1 | 1 |
|  |  | KRV vs Anakinra | 0.008 | 0.032 |
|  |  | KRV vs KRV + ITF | 1 | 1 |
|  |  | KRV vs KRV + Anakinra | 1 | 1 |
|  |  | Uninfected vs ITF | 1 | 1 |
|  |  | Uninfected vs Anakinra | 0.008 | 0.032 |
|  |  | Uninfected vs KRV + ITF | 1 | 1 |
|  |  | Uninfected vs KRV + Anakinra | 1 | 1 |
|  |  | Uninfected vs KRV | 1 | 1 |
|  |  | KRV + Anakinra vs KRV + ITF | 1 | 1 |
| Ruminococcaceae | 0.044 | ITF vs KRV + ITF | 0.674 | 0.8 |
|  |  | Anakinra vs ITF | 0.279 | 0.426 |
|  |  | Anakinra vs KRV + Anakinra | 0.691 | 0.818 |
|  |  | KRV vs ITF | 0.006 | 0.025 |
|  |  | KRV vs Anakinra | 0.08 | 0.166 |
|  |  | KRV vs KRV + ITF | 0.015 | 0.049 |
|  |  | KRV vs KRV + Anakinra | 0.17 | 0.297 |
|  |  | Uninfected vs ITF | 0.022 | 0.066 |
|  |  | Uninfected vs Anakinra | 0.217 | 0.354 |
|  |  | Uninfected vs KRV + ITF | 0.054 | 0.122 |
|  |  | Uninfected vs KRV + Anakinra | 0.396 | 0.545 |
|  |  | Uninfected vs KRV | 0.59 | 0.725 |
|  |  | KRV + Anakinra vs KRV + ITF | 0.276 | 0.424 |
| Bacteroidales | 0.045 | ITF vs KRV + ITF | 0.006 | 0.026 |
|  |  | Anakinra vs ITF | 0.405 | 0.553 |
|  |  | Anakinra vs KRV + Anakinra | 0.068 | 0.146 |
|  |  | KRV vs ITF | 0.069 | 0.147 |
|  |  | KRV vs Anakinra | 0.324 | 0.48 |
|  |  | KRV vs KRV + ITF | 0.34 | 0.494 |
|  |  | KRV vs KRV + Anakinra | 0.381 | 0.539 |
|  |  | Uninfected vs ITF | 0.177 | 0.304 |
|  |  | Uninfected vs Anakinra | 0.61 | 0.74 |
|  |  | Uninfected vs KRV + ITF | 0.151 | 0.272 |
|  |  | Uninfected vs KRV + Anakinra | 0.179 | 0.306 |
|  |  | Uninfected vs KRV | 0.63 | 0.757 |
|  |  | KRV + Anakinra vs KRV + ITF | 0.963 | 1 |
| S24-7 | 0.045 | ITF vs KRV + ITF | 0.232 | 0.372 |
|  |  | Anakinra vs ITF | 0.007 | 0.03 |
|  |  | Anakinra vs KRV + Anakinra | 0.006 | 0.025 |
|  |  | KRV vs ITF | 0.668 | 0.795 |
|  |  | KRV vs Anakinra | 0.025 | 0.071 |
|  |  | KRV vs KRV + ITF | 0.466 | 0.613 |
|  |  | KRV vs KRV + Anakinra | 0.533 | 0.67 |
|  |  | Uninfected vs ITF | 0.99 | 1 |
|  |  | Uninfected vs Anakinra | 0.009 | 0.035 |
|  |  | Uninfected vs KRV + ITF | 0.245 | 0.389 |
|  |  | Uninfected vs KRV + Anakinra | 0.842 | 0.943 |
|  |  | Uninfected vs KRV | 0.671 | 0.797 |
|  |  | KRV + Anakinra vs KRV + ITF | 0.174 | 0.302 |
| Collinsella | 0.046 | ITF vs KRV + ITF | 1 | 1 |
|  |  | Anakinra vs ITF | 1 | 1 |
|  |  | Anakinra vs KRV + Anakinra | 0.463 | 0.61 |
|  |  | KRV vs ITF | 0.01 | 0.038 |
|  |  | KRV vs Anakinra | 0.013 | 0.044 |
|  |  | KRV vs KRV + ITF | 0.01 | 0.038 |
|  |  | KRV vs KRV + Anakinra | 0.068 | 0.146 |
|  |  | Uninfected vs ITF | 0.078 | 0.161 |
|  |  | Uninfected vs Anakinra | 0.089 | 0.179 |
|  |  | Uninfected vs KRV + ITF | 0.078 | 0.161 |
|  |  | Uninfected vs KRV + Anakinra | 0.318 | 0.473 |
|  |  | Uninfected vs KRV | 0.386 | 0.541 |
|  |  | KRV + Anakinra vs KRV + ITF | 0.446 | 0.598 |
